# Sodium orthovanadate overcomes sorafenib resistance of hepatocellular carcinoma cells by inhibiting Na^+^/K^+^-ATPase activity and hypoxia-inducible pathways

**DOI:** 10.1038/s41598-018-28010-y

**Published:** 2018-06-26

**Authors:** Wenjing Jiang, Guangxin Li, Weidong Li, Ping Wang, Peng Xiu, Xian Jiang, Bing Liu, Xueying Sun, Hongchi Jiang

**Affiliations:** 1 Department of General Surgery, the First Affiliated Hospital of Harbin Medical University, Harbin, 150001 China; 2 Department of General Surgery, Qianfoshan Hospital Affiliated to Shandong University, Jinan, 250014 China; 3 The Hepatosplenic Surgery Center, the First Affiliated Hospital of Harbin Medical University, Harbin, 150001 China; 4 Department of Interventional Radiology, The Third Affiliated Hospital of Harbin Medical University, Harbin, 150081 China

## Abstract

The resistance to sorafenib highly affects its clinical benefits for treating hepatocellular carcinoma (HCC). Sodium orthovanadate (SOV) is a phosphate analog that displays anti-cancer activities against various types of malignancies including HCC. The present study has demonstrated that SOV is able to overcome sorafenib resistance and strengthens sorafenib in suppressing sorafenib-resistant HCC cells *in vitro* and in animal models. Similar to its action on parental HCC cells, SOV induced cell cycle arrest at G2/M phases by regulating cyclin B1 and cyclin-dependent kinase 1, and apoptosis by reducing mitochondrial membrane potential, in sorafenib-resistant HCC cells. More importantly, SOV inhibited ATPase activity, which was significantly elevated in sorafenib-resistant HCC cells. SOV also reduced the expression of HIF-1α and HIF-2α and their nuclear translocation, resulting in downregulation of their downstream factors including vascular endothelial growth factor, lactate dehydrogenase-A and glucose transporter 1. Its ability to inhibit ATPase activity and hypoxia-inducible pathways enabled SOV to efficiently suppress both normoxic and hypoxic cells, which compose cancer cell populations inside sorafenib-resistant HCC tumors. The present results indicate that SOV may be a potent candidate drug for overcoming the resistance to sorafenib in treating HCC.

## Introduction

Hepatocellular carcinoma (HCC) remains the third leading cause of cancer mortality worldwide^1^. Sorafenib is a globally accepted systemic drug, which prolongs the overall survival of patients with advanced HCC for only 2–3 months^2,3^. Particularly, the acquired resistance to sorafenib greatly limits its beneficial effects^4^. What’s worse, inhibition of the molecules and pathways activated in sorafenib-resistant HCC (SR-HCC) cells leads to the bypass activation of compensatory loops^5^, indicating that the mechanisms underlying sorafenib resistance are highly complex. Therefore, further exploring the mechanisms and seeking agents for overcoming this resistance continue to be a hotspot of research on HCC^6^.

Na^+^/K^+^-ATPase, a transmembrane protein, was originally described by Skou, a Nobel laureate, in 1957^7^. It translocates sodium and potassium ions across the cell membrane utilizing ATP as the driving force^8^. Recently, the potential involvement of Na^+^/K^+^-ATPase in a growing number of cancers has drawn attention by many researchers since it is abnormally expressed and displays multiple functions in cancer cells^7^. More importantly, many lines of studies have demonstrated that Na^+^/K^+^-ATPase play key roles in drug resistance of cancer cells by triggering intracellular signaling^9^. Higher ATPase activity has been observed in drug-resistant cancer cells^10^. Inhibition of Na^+^/K^+^-ATPase re-sensitized multiple cancer cells to various chemotherapeutic drugs^8,11–14^. However, it has not been investigated whether Na^+^/K^+^-ATPase is involved in the sorafenib resistance of HCC.

Sodium orthovanadate (SOV), a phosphate analog, has exhibited activities in inhibiting protein tyrosine phosphatases and ATPases^15^. SOV effectively inhibits certain plasma membrane ATPases including Na^+^/K^+^-ATPase, but not other ATPases^16^. SOV has exhibited anti-cancer activities against several types of cancer experimentally^17–20^. We have previously reported that SOV suppresses the growth of HCC cells in culture and in an orthotopic mouse model^21^. Although its molecular mechanisms remain unclear, SOV induces cell cycle arrest at G2/M phase and programmed cell death of cancer cells^21,22^. However, it is unknown whether it also displays inhibitory activities against SR-HCC cells.

It is well known that tumor hypoxia induces cancer drug resistance by activating hypoxic pathways, which are controlled by hypoxia-inducible factors (HIFs)^23,24^. Complex with HIF-1β (also known as aryl hydrocarbon receptor nuclear translocator [ARNT]), HIF-1α and HIF-2α each subunit can form a heterodimer that binds hypoxia-response elements (HREs) in the promoters of the targeted genes^24^. We and others have demonstrated that HIF-1α and HIF-2α participate in the resistance to pharmacological drugs including sorafenib^25–27^. Inhibition of HIFs improves the response of resistant hypoxic HCC cells to sorafenib^27,28^. In addition, Na^+^/K^+^-ATPase inhibitors are able to downregulate the expression of HIF-1α in cancer cells^29,30^. Therefore, it can be speculated that SOV as an ATPase inhibitor may also inhibit HIF pathways in SR-HCC cells.

## Results

### Increased ATPase activity contributes to sorafenib resistance in HCC cells

Two SR-HCC cell lines, HepG2-SR and Huh7-SR, were established from sorafenib-sensitive human HCC HepG2 and Huh7 cells, respectively. They were shown to be more insensitive to sorafenib-induced growth inhibition (Fig. S1a) and apoptosis (Fig. S1b) *in vitro* than the respective parental cells, in agreement with our previous studies^31,32^.

**Figure 1:**
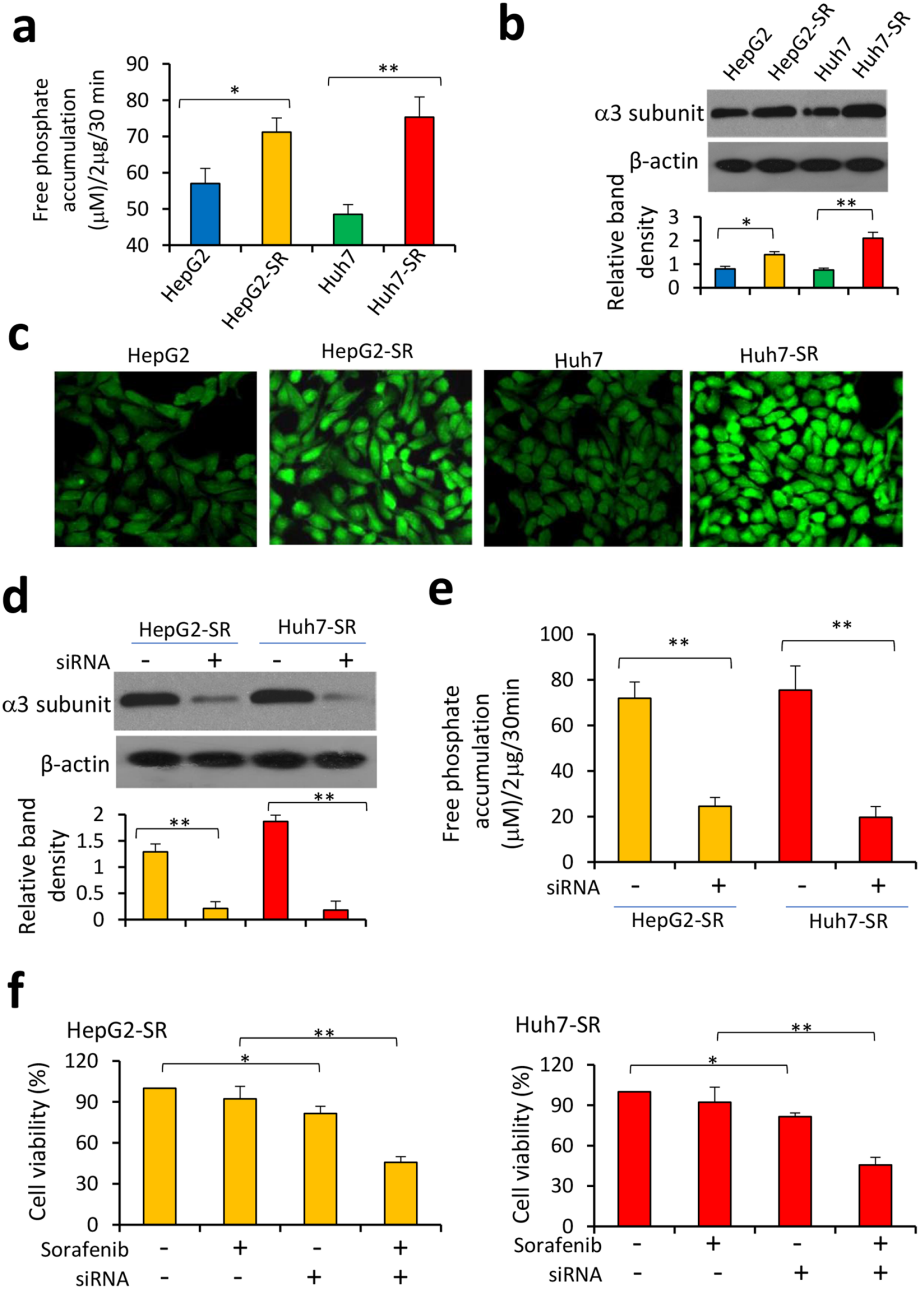
Increased ATPase activity contributes to sorafenib resistance of HCC cells. (**a**) HepG2, HepG2-SR, Huh7 and Huh7-SR cells were lysed for measuring ATPase activity, which was represented by the amount of phosphate release from cells by using malachite green reagent. (**b**,**c**) The expression of Na^+^/K^+^-ATPase α3 subunit protein in the above cells was detected by immunoblotting (**b**) and immunocytochemistry (**c**). (**d**,**e**) HepG2-SR and Huh7-SR cells were transfected with control siRNA or siRNA targeting the α3 subunit. Cells were harvested 48 h later, and then subjected to immunoblotting (**d**) and ATPase activity assays (**e**). (**f**) Cells transfected with control siRNA or siRNA targeting the α3 subunit were incubated for 48 h in the presence or absence of sorafenib (5 μM). Cell viability (%) was measured. The density of each immunoblotting band was normalized to β-actin. “*” Indicates P < 0.05, and “**” P < 0.001.

It has been reported that drug-resistant cancer cells possess higher ATPase activity^10,13^. In accord, ATPase activity was significantly higher in HepG2-SR and Huh7-SR cells than in their respective parental cells (Fig. 1a). We next detected the expression of six potential Na^+^/K^+^-ATPase subunit mRNAs, including *ATP1A1*, *ATP1A2*, *ATP1A3*, *ATP1A4*, *ATP1B1* and *ATP1B2*. The expression level of *ATP1A3* mRNA was significantly higher in HepG2-SR and Huh7-SR cells than in the respective parental cells; while the expression levels of the other miRNAs remained unchanged (Fig. S2). The results were in consistence the expression level of Na^+^/K^+^-ATPase α3 subunit, the encoding protein of *ATP1A3* gene, detected by immunoblotting (Fig. 1b) and immunocytochemistry (Fig. 1c). Furthermore, transfection of siRNA targeting Na^+^/K^+^-ATPase α3 subunit downregulated its expression (Fig. 1d) and significantly reduced ATPase activity in SR-HCC cells (Fig. 1e). Depletion of α3 subunit also re-sensitized SR-HCC cells to sorafenib-induced growth inhibition (Fig. 1f).

### SOV reduces ATPase activity in sorafenib-resistant HCC cells

**Figure 2:**
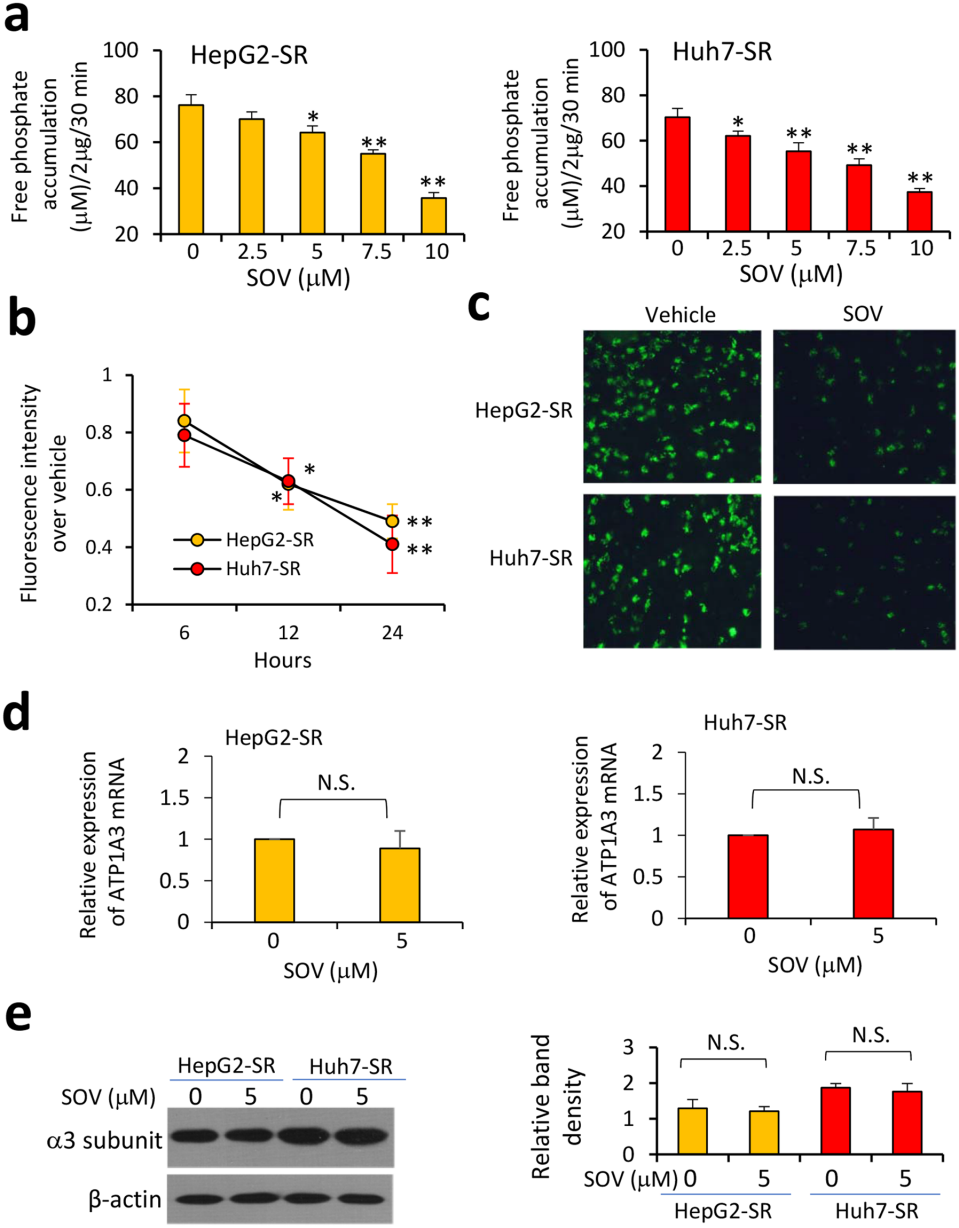
Sodium orthovanadate (SOV) reduces ATPase activity in sorafenib-resistant HCC cells. (**a**) HepG2-SR and Huh7-SR cells were incubated for 30 min with SOV at various concentrations. Cells were harvested and lysed, and the amounts of phosphate release were measured. (**b**,**c**) Cells were incubated with vehicle or SOV at 5 μM for 6, 12 or 24 h, and then stained with K^+^ fluorescent dye PBFI-AM. (**b**) Fluorescence intensity was measured. (**c**) Representative images were taken from PBFI-AM-stained cells incubated with vehicle or SOV (5 μM) for 24 h. (**d**,**e**) Cells were incubated with 5 μM of SOV for 24 h, and then subjected to qRT-PCR for measuring the expression of Na^+^/K^+^-ATPase α3 subunit mRNA (**d**) and immunoblotting for its protein expression (**e**). The density of each immunoblotting band was normalized to β-actin. “N.S.” indicates no significance. “*” (P < 0.05) and “**” (P < 0.001) vs. vehicle-treated cells.

SOV is an accepted ATPase inhibitor^16^, thus incubation of SOV led to significantly lower levels of ATPase activity as measured free phosphate accumulation in SR-HCC cells in a concentration-dependent way (Fig. 2a). To support the results, we measured intracellular contents of K^+^, representing the ion transporting function of Na^+^/K^+^-ATPase. Cells were incubated with 5 μM of SOV and then stained with a K^+^ fluorescent dye, PBFI-AM. SOV incubation led to a significant reduction of intracellular K^+^ contents in SR-HCC cells in a time-dependent manner (Fig. 2b,c). However, SOV had little effects on the expression of either mRNA (Fig. 2d) or protein of Na^+^/K^+^-ATPase α3 subunit (Fig. 2e).

### SOV inhibits the proliferation of SR-HCC cells by inducing cell cycle arrest at G2/M phases

**Figure 3:**
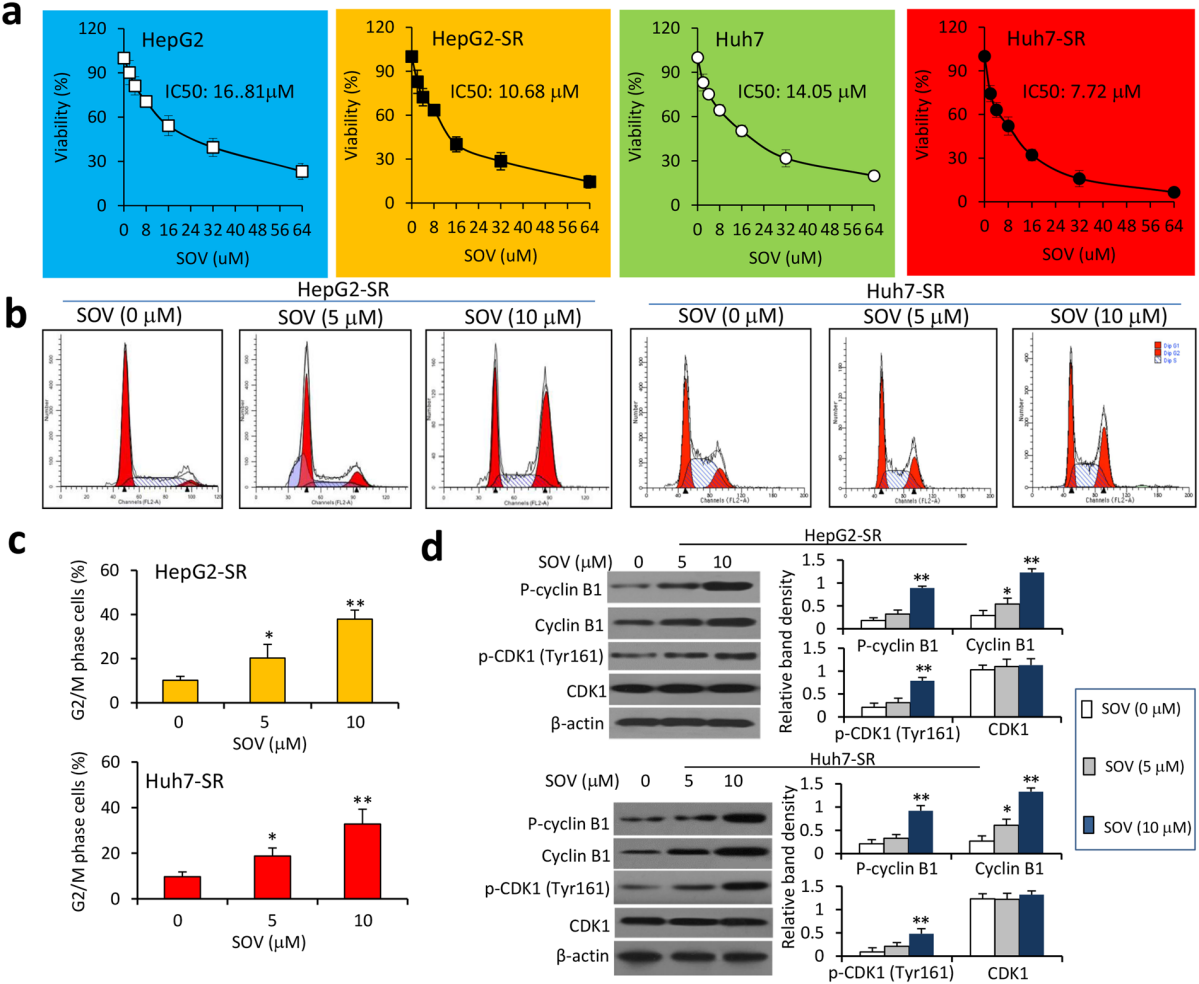
SOV inhibits the proliferation of HCC cells. HepG2, HepG2-SR, Huh7 and Huh7-SR cells were incubated for 48 h with various concentrations of SOV. (**a**) Cell viability (%) was compared with respective untreated cells. With a logarithmic regression analysis, the values of IC_50_ for each cell type were calculated. (**b**) HepG2-SR and Huh7-SR cells treated with SOV at concentrations of 0, 5 and 10 μM were cytometrically analyzed for determining cell cycle distribution, and representative histograms are shown. (**c**) The percentages of cells arrested at G2/M phases were plotted. (**d**) Cells were subjected to immunoblotting. The density of each immunoblotting band was normalized to β-actin. “*” (P < 0.05) and “**” P < 0.001 vs. vehicle-treated cells.

We have previously reported that SOV inhibits the proliferation of HCC cells^21^. Here, we could further confirm its proliferation-inhibiting activities on four HCC cell lines, HepG2, Huh7, SK-Hep-1 and Hep3B cells, in a concentration-dependent way (Fig. S3a). More importantly, SOV displayed a stronger activity in inhibiting SR-HCC cells than the respective parental cells (Fig. 3a). Thus, the value of IC_50_ for HepG2 cells was 16.81 μM, which was significantly higher than that for HepG2-SR cells (10.68 μM); the values of IC_50_ for Huh7 cells was 14.05 μM, significantly higher than that of Huh7-SR cells (7.72 μM) (Fig. 3A). Incubation of SOV resulted in cell cycle arrest at G2/M phases in SR-HCC cells (Fig. 3b,c). In exploring the mechanism, we found that SOV increased the expression of cyclin B1 and its phosphorylated form, and phosphorylated cyclin-dependent kinase 1 (CDK1) at Tyr161, but had little effect on CDK1 expression. However, this effect of SOV seems to be independent of its inhibitory activity on Na^+^/K^+^-ATPase, since depletion of Na^+^/K^+^-ATPase α3 subunit did not significantly induce cell cycle arrest at G2/M phases (Fig. S4a) or alter the expression of cyclin B1 and CDK1 or their phosphorylated forms (Fig. S4b). Inhibition of Na^+^/K^+^-ATPase with ouabain, a well-known Na^+^/K^+^-ATPase inhibitor^13^, did not induce cell cycle arrest at G2/M phases, either (Fig. S4c).

### SOV promotes apoptosis and reduces mitochondrial membrane potential of SR-HCC cells

**Figure 4:**
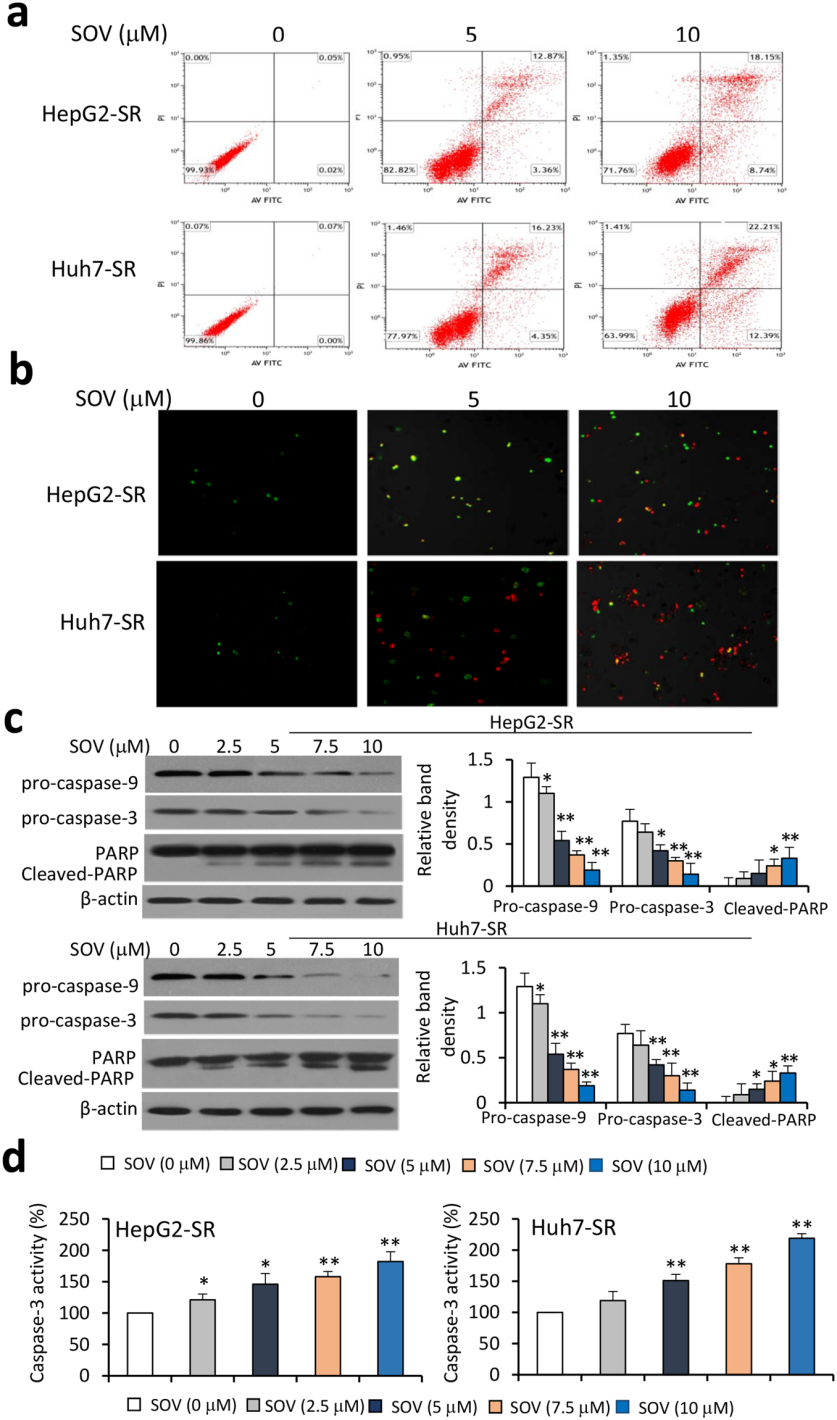
SOV induces apoptosis of sorafenib-resistant HCC cells. HepG2-SR and Huh7-SR cells were incubated for 48 h with various concentrations of SOV. (**a**) Apoptosis rates (%) were measured, and representative histograms of cytometrically analyzed cells are shown. (**b**) Representative images were taken from cells stained with Annexin V/propidium iodide. (**c**) Cells were subjected to immunoblotting. The density of each immunoblotting band was normalized to β-actin. (**d**) Cells were analyzed for measuring the activity of caspase-3. “*” (P < 0.05) and “**” (P < 0.001) vs. vehicle-treated cells.

Incubation of SOV increased apoptosis rates of HepG2, Huh7, SK-Hep-1 and Hep3B cells in a dose-dependent manner (Fig. S3b), in agreement with our previous report^21^. Furthermore, SOV exhibited a stronger pro-apoptotic activity on SR-HCC cells than on their respective parental cells (Fig. S5 and Fig. 4a,b). SOV increased the cleavage of caspase-9 and caspase-3, resulting in reduced expression of pro-caspase-9 and pro-caspase-3 in HepG2-SR and Huh7-SR cells in a dose-dependent manner (Fig. 4c), which was supported by the pattern of caspase-3 activity (Fig. 4d). SOV also increased the cleavage of Poly (ADP-ribose) polymerase (PARP) in a dose-dependent manner (Fig. 4c). The results indicate that SOV may induce the apoptosis of SR-HCC cells via caspase-dependent and -independent pathways. Mitochondrial dysfunction participates in the induction of apoptosis and is a central to apoptotic pathways^21^. Incubation of SOV significantly diminished mitochondrial membrane potential (ΔΨM) of HepG2-SR and Huh7-SR cells (Fig. S6), in consistence with its actions on parental HCC cells^21^.

### SOV enhances the anti-cancer activity of sorafenib against SR-HCC cells *in vitro*

The above results suggested that SOV might be able to strengthen sorafenib in suppressing SR-HCC cells. HepG2-SR and Huh7-SR cells were incubated with sorafenib (2.5 μM), SOV (5 μM) or their combination. Sorafenib alone only slightly reduced the viability and increased the apoptosis of SR-HCC cells; but SOV significantly reduced the viability and increased the apoptosis of SR-HCC cells (Fig. S7). The combination of SOV and sorafenib displayed stronger proliferation-inhibitory (Fig. S7a) and pro-apoptotic (Fig. S7b) activities than either SOV or sorafenib alone. We further calculated coefficient of drug interaction (CDI) as described previously^27,31^. The values of CDI were 0.67 and 0.63 in HepG2-SR and Huh7-SR cells, respectively, indicating SOV and sorafenib worked synergistically in inducing apoptosis of SR-HCC cells (Fig. S7b).

### SOV inhibits hypoxia-inducible pathways in SR-HCC cells

**Figure 5:**
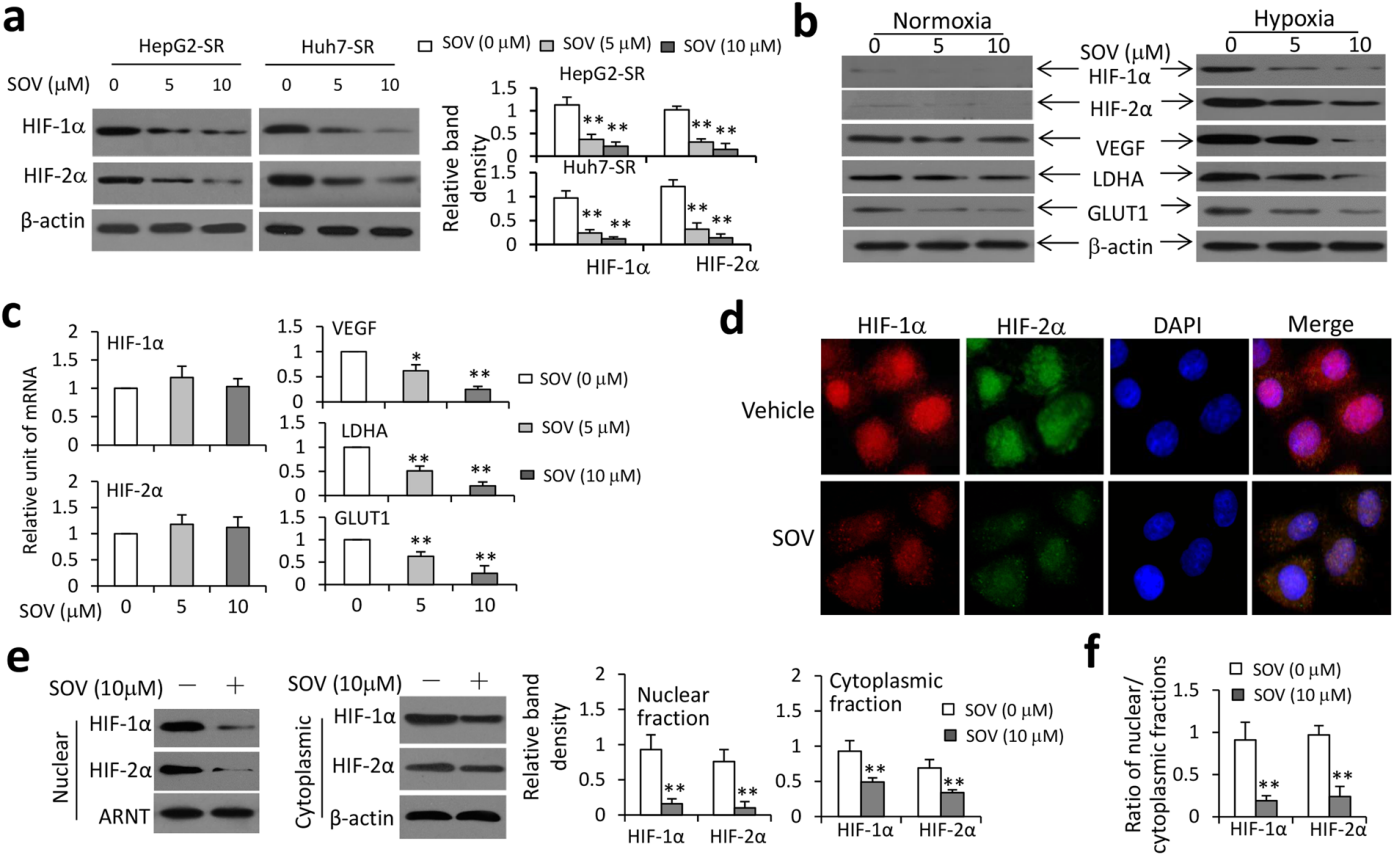
SOV inhibits the expression of HIF-1α and HIF-2α proteins and their nuclear translocation in sorafenib-resistant HCC cells. (**a**) HepG2-SR and Huh7-SR cells were incubated with SOV (0, 5 or 10 μM) under hypoxia (1% O_2_) for 24 h. Cells were lysed and immunoblotted. Band density was normalized to β-actin. (**b**,**c**) Huh7-SR cells were incubated with SOV (0, 5 or 10 μM) under normoxia or hypoxia (1% O_2_) for 24 h, and then subjected to immunoblotting (**b**). (**c**) Hypoxic Huh7-SR cells were subjected to qRT-PCR for detecting the expression of mRNAs. The level of mRNA from untreated cells was defined as 1. (**d**) Huh7-SR cells incubated with vehicle or SOV (10 μM) under hypoxia (1% O_2_) for 24 h, and then immunostained with Abs against HIF-1α (red), HIF-2α (green) and DAPI (cellular nuclei, blue). (**e**) The nuclear and cytoplasmic fractions of vehicle- or SOV (10 μM)-treated hypoxic Huh7-SR cells were immunoblotted. The band density of HIF-1α or HIF-2α protein in nuclear fractions was normalized to ARNT, and that in cytoplasmic fractions, β-actin. (**f**) The ratio of HIF-1α or HIF-2α protein in nuclear/cytoplasmic fractions was calculated. “*” (P < 0.05) and “**” (P < 0.001) indicate a significant difference from respective vehicle-treated cells.

It has been reported that Na^+^/K^+^-ATPase inhibitors reduces HIF-1α protein synthesis and the expression of HIF-1 target genes in cancer cells^29,30^. Here we showed that SOV downregulated the expression of HIF-1α and HIF-2α proteins in hypoxic SR-HCC cells in a concentration-dependent way (Fig. 5a). SOV also inhibited the expression of VEGF, LDHA and GLUT1, the downstream factors of HIF pathways^24^, in hypoxic Huh7-SR cells, but had a weaker effect on normoxic cells (Fig. 5b). However, SOV had little effect on the expression of HIF-1α and HIF-2α mRNAs, but could induce the downregulation of VEGF, LDHA and GLUT1 mRNAs (Fig. 5c), indicating that SOV may regulate HIF-1α and HIF-2α at posttranslational levels. Its ability to downregulate HIF-1α and HIF-2α proteins was confirmed by immunocytochemistry (Fig. 5d). We next investigated whether SOV could influence the nuclear translocation of HIF-1α or HIF-2α. Cells were lysed to isolate nuclear and cytoplasmic fractions, which were separately immunoblotted. SOV incubation lead to a significant reduction in nuclear and cytoplasmic expression of HIF-1α and HIF-2α proteins and decreased the nuclear/cytoplasmic fraction ratio of HIF-1α or HIF-2α protein (Fig. 5e,f). In addition, the ability of SOV to inhibit HIF seems to be dependent on its inhibitory effect on ATPase, since depletion of Na^+^/K^+^-ATPase α3 subunit also led to downregulation of HIF-1α and HIF-2α proteins (Fig. S8).

### SOV enhances the effect of sorafenib in inhibiting proliferation and inducing apoptosis of hypoxic SR-HCC cells

**Figure 6:**
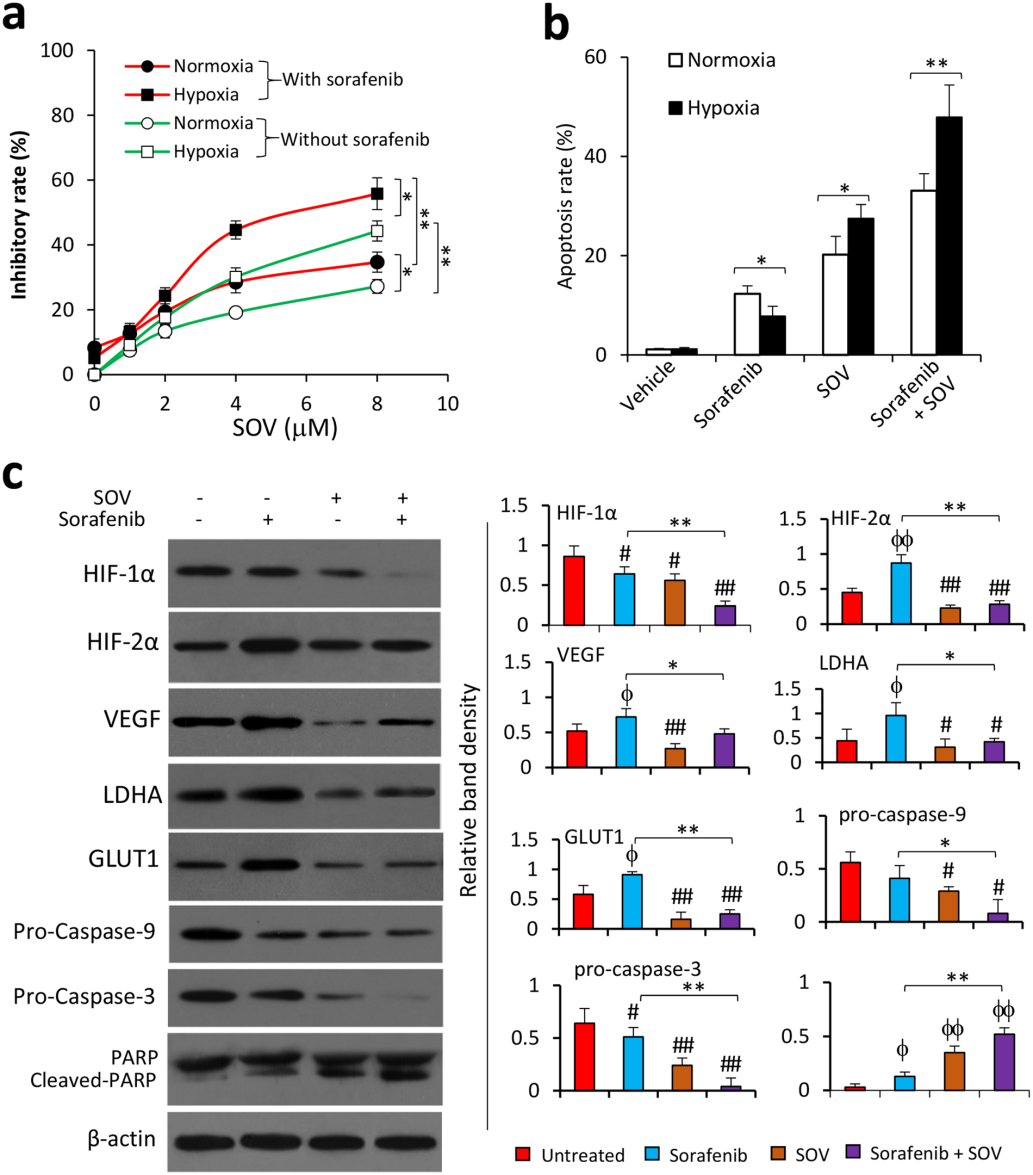
SOV synergizes with sorafenib to inhibit the proliferation and induce the apoptosis of hypoxic sorafenib-resistant HCC cells. (**a**) Huh7-SR cells were incubated for 24 h with serial concentrations of SOV (0, 1, 2, 4 or 8 μM) in the absence or presence of sorafenib (2.5 μM) under normoxia or hypoxia (1% O_2_) for 24 h. Cell viability was assessed and the inhibitory rate (%) was calculated. (**b**,**c**) Huh7-SR cells were incubated for 24 h with vehicle, or sorafenib (2.5 μM), or SOV (2 μM), or the combination of sorafenib and SOV under normoxia or hypoxia (1% O_2_). Cells were subjected to flow cytometry for analyzing apoptosis (**b**) and immunoblotting (**c**). Band densities were normalized to β-actin. “*” Indicates P < 0.05, and “**” P < 0.001. ^“#”^ (P < 0.05) and ^“##”^ (P < 0.001) indicate a significant reduction, ^“ϕ”^ (P < 0.05) and ^“ϕϕ”^ (P < 0.001), a significant increase, from respective vehicle-treated cells.

SOV increased the proliferation inhibitory activities of sorafenib on both normoxic and hypoxic Huh7-SR cells in a concentration-dependent way, and hypoxic cells showed more sensitive to SOV-induced proliferation inhibition (Fig. 6a). Similarly, compared to normoxic cells, hypoxic cells showed more insensitive to sorafenib, but were more sensitive to SOV-induced apoptosis (Fig. 6b). Sorafenib and SOV worked together to induce more apoptosis than either sorafenib or SOV alone, in both normoxic and hypoxic cells (Fig. 6b). The values of CDI were 0.955 and 0.67 in normoxic and hypoxic Huh7-SR cells treated with SOV and sorafenib, respectively, indicating that sorafenib and SOV worked synergistically in hypoxic but not normoxic cells. SOV enhanced sorafenib in downregulating HIF-1α expression, and amended the elevated expression of HIF-2α, VEGF, LDHA and GLUT1, which was caused by sorafenib (Fig. 6c). SOV also increased the effect of sorafenib to induce the cleavage of caspase-9, caspase-3 and PARP (Fig. 6c).

### SOV administration suppresses sorafenib-resistant tumors *in vivo*

**Figure 7:**
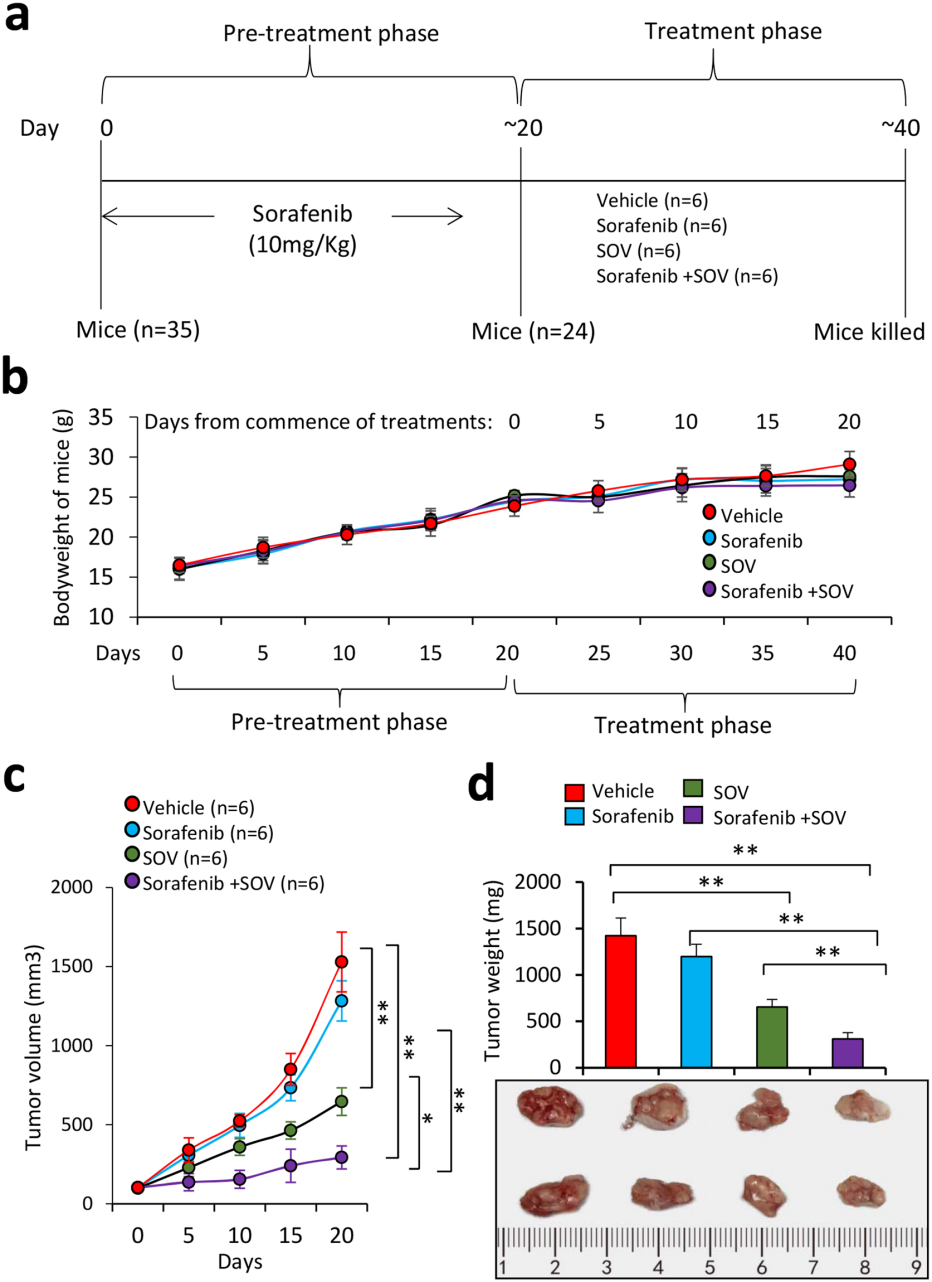
SOV strengthens sorafenib in suppressing sorafenib-resistant tumors *in vivo*. (**a**) Animal experimental schedule was described in Materials and Methods. (**b**) The bodyweights of mice were monitored. (**c**) The size (mm^3^) of tumors was recorded. (**d**) Tumors harvested at the end of experiments were weighed and representative tumors photographed. “*” Indicates P < 0.05, and “**” P < 0.001.

A group of 35 mice underwent subcutaneous injections of Huh7-SR cells (5 × 10^6^) and received daily oral sorafenib at 10 mg/kg, which was used to maintain the sorafenib-resistant capacity of Huh7-SR cells in mice^31,32^. Twenty days later, 24 mice (24/35) bearing palpable tumors of ~100 mm^3^ in volume were randomly assigned to four groups, which received administration of vehicle, sorafenib, SOV or sorafenib plus SOV, respectively (Fig. 7a). Treatments with sorafenib, SOV or sorafenib plus SOV had almost no effect on bodyweight of mice (Fig. 7b). SR-HCC tumors were shown to be resistant to sorafenib *in vivo* since tumors treated with sorafenib (1283.41 ± 135.84 mm^3^ in volume, 978.4 ± 135.6 mg in weight) were only slightly smaller than those treated with vehicle (1529.22 ± 195.31 mm^3^ in volume, 1423.8 ± 189.5 mg in weight), 20 days after the commencement of treatments (Fig. 7c,d), in agreement with our previous study^31,32^. However, SOV treatment significantly suppressed tumors (646.25 ± 87.3 mm^3^ in volume, 653.7 ± 82.4 mg in weight) by 54.1%, and the combination therapy resulted in a further reduction of tumors (294.38 ± 72.65 mm^3^ in volume, 311.3 ± 68.5 mg in weight) by 77.5%, compared with control tumors, 20 days after the commencement of treatments (Fig. 7c,d).

The above tumors were further analyzed for detecting cell proliferation and apoptosis *in situ* (Fig. S9a,b). Again, sorafenib had a weak effect in inhibiting cell proliferation and also a weak pro-apoptotic activity against sorafenib-resistant tumors; while SOV significantly reduced cell proliferation and apoptosis *in situ*. SOV also strengthened the effects of sorafenib in inhibiting cell proliferation and promoting apoptosis, since tumors treated with the combinational therapy had a further reduction in proliferation index and a further increase in apoptosis rate, compared with either sorafenib or SOV monotherapy. Sorafenib or SOV alone also reduced tumoral microvessel density, and the combinational therapy led to even less microvessel density (Fig. S9a,b). Tumoral expression of HIF-1α and HIF-2α as detected by immunohistochemistry (Fig. S9a) was in consistent with the *in vitro* results (Fig. 6c). Immunoblotting of tumor tissues (Fig. S9c) also showed similar patterns in the expression of HIF-1α, HIF-2α, VEGF, pro-caspase-9, pro-caspase-3 and cleaved PARP, compared with the *in vitro* results (Fig. 6c).

## Discussion

The present results have revealed that SOV overcomes sorafenib resistance and strengthens sorafenib in suppressing SR-HCC cells *in vitro* and *in vivo*. Similar to its action on parental HCC cells, SOV induced cell cycle arrest at G2/M phases by regulating cyclin B1 and CDK1, and apoptosis by reducing mitochondrial membrane potential, in SR-HCC cells. More importantly, SOV inhibited ATPase activity, which was significantly elevated in SR-HCC cells. SOV also reduced the expression of HIF-1α and HIF-2α and their nuclear translocation, leading to downregulation of their downstream factors including VEGF, LDHA and GLUT1. Its ability to inhibit ATPase activity and hypoxia-inducible pathways rendered hypoxic cells to be more sensitive to SOV, and enabled SOV to suppress both normoxic and hypoxic cells, which compose the cancer cell populations inside SR-HCC tumors. In addition, the anti-cancer activity of SOV to overcome sorafenib resistance of HCC cells has been confirmed in experimental animals. Since the acquired resistance to sorafenib particularly limits its clinical benefits^4^, we established a sorafenib-resistant HCC animal model by inoculating HCC cells that had acquired the resistance to sorafenib in culture and administering mice with low dose of sorafenib. The use of low dose of sorafenib was to maintain the sorafenib-resistant ability of SR-HCC cells *in situ*^31,32^.

A physiological activity of ATPases, particularly Na^+^/K^+^-ATPase, is necessary for maintaining normal cellular biology, and abnormal activity of Na^+^/K^+^-ATPase is involved in the biological behavior of cancer cells^7^. Enhanced ATPase activity contributes to the biological behavior of cancer cells and the mechanisms for drug resistance of cancer cells^33^, and Na^+^/K^+^-ATPase activity is elevated in drug-resistant cancer cells^9,10^. Blocking Na^+^/K^+^-ATPase by inhibitors or gene knockdown re-sensitized cancer cells to chemotherapy^8,10–14^. In accord, we have shown herein that SR-HCC cells had higher Na^+^/K^+^-ATPase activity. Na^+^/K^+^-ATPase is functionally composed of catalytic α and regulatory β subunits, and an optional γ subunit^34^. The catalytic α subunit can be subclassified into 4 different tissue-specific isoforms, namely α1, α2, α3 and α4. The regulatory β subunits including β1 and β2 are ubiquitously expressed in mammalian cells^35^. The present results showed that SR-HCC cells overexpressed the Na^+^/K^+^-ATPase α3 subunit and its depletion re-sensitized SR-HCC cells. Inhibition of ATPase by SOV also re-sensitized SR-HCC cells, but had little effects on the expression of the α3 subunit, indicating that its inhibitory activity on Na^+^/K^+^-ATPase mainly rely on modulating the oligomerization of ATPase^36^. However, the exact mechanism for its effects on ATPase inhibition needs further investigation.

Cyclin B1 and CDK1 are key molecules in regulating cell cycle progress since cyclin B1 forms a molecular complex with phosphorylated CDK1 to control the transition of cells from S phase to G2/M phase^37^. In agreement with previous studies^21,22^, the present study has demonstrated that SOV increased the expression of cyclin B1 and phosphorylated cyclin B1, and phosphorylated CDK1 at Tyr161, in SR-HCC cells. The phosphorylation of the conserved tyrosine (Tyr15 in humans) is thought to alter ATP orientation, preventing efficient kinase activity^37^. However, this effect might be independent of Na^+^/K^+^-ATPase since depletion of Na^+^/K^+^-ATPase α3 subunit did not significantly induce cell cycle arrest at G2/M phases.

It is well accepted that hypoxic cancer cells feature resistance to chemotherapies since they overexpress HIFs that enable them to be adapted to hypoxia^23^. Thus hypoxia-inducible pathway has emerged a potential target for developing anti-cancer drugs, which are able to inhibit the accumulation and/or activity of HIFs^38^. In support of previous reports^26,27^, the present study has shown that sorafenib inhibited the expression of HIF-1α and increased HIF-2α expression. SOV reduced the expression of both HIF-1α and HIF-2α, and their nuclear translocation, leading to downregulation of VEGF, LDHA and GLUT1, the downstream factors of hypoxia-inducible pathways^24^. The abnormal microvasculature can cause hypoxic regions of tumor tissues, and oxygen partial pressure varies tissues because of oxygen diffusing range, resulting in the co-existence of hypoxic and normoxic cell populations inside solid tumors^39^. Therefore, SOV is able to suppress both normoxic and hypoxic cancer cells, particularly hypoxic SR-HCC cells, which are extremely resistant to sorafenib.

**Figure 8:**
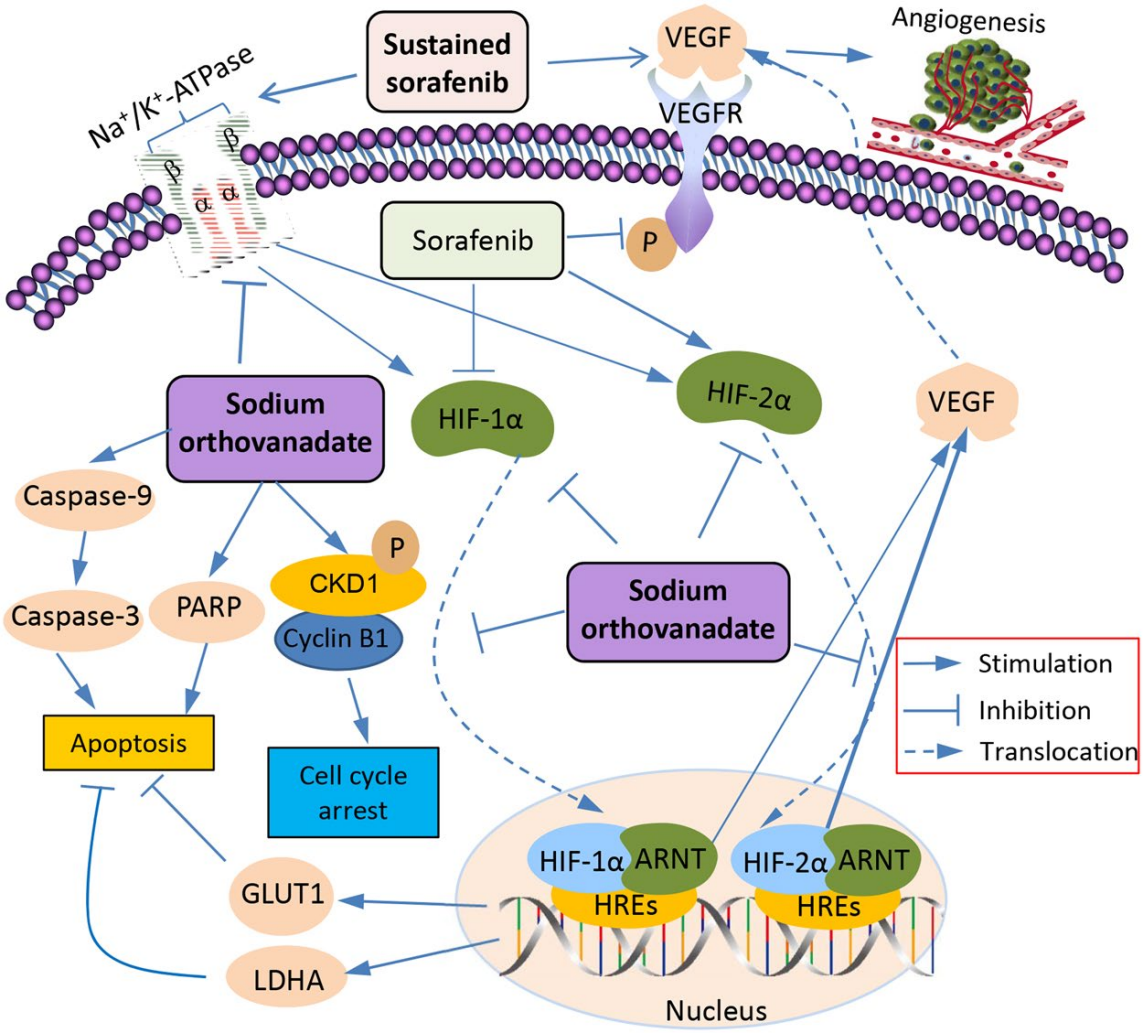
A schematic diagram of proposed mechanisms by which sodium orthovanadate displays its ability to enhance the effects of sorafenib against sorafenib-resistant HCC cells. Abbreviations: ARNT, aryl hydrocarbon receptor nuclear translocator; CDK1, cyclin-dependent kinase 1; HIF-1α, hypoxia-inducible factor-1α; HIF-2α, hypoxia-inducible factor-2α; GLUT1, glucose transporter 1; HREs, hypoxia-response elements; LDHA, lactate dehydrogenase-A; PARP, poly (ADP-ribose) polymerase; VEGF, vascular endothelial growth factor; VEGFR, vascular endothelial growth factor receptor.

The investigated mechanisms accounting for how SOV enhances the effects of sorafenib in suppressing SR-HCC cells are depicted in Fig. 8. Sorafenib inhibits tumor angiogenesis by targeting VEGFR^2^, but sustained sorafenib also increases the production of VEGF through its effects on HIFs. Sorafenib treatment leads to an increased expression of HIF-2α protein through compensatory mechanism by reducing the synthesis of HIF-1α protein^26,27^. HIF-1α and HIF-2α subunits complex with ARNT to form HIF-1 and HIF-2, respectively, and translocate to the nucleus^24^, where they regulate VEGF, LDHA and GLUT1 by binding to HREs in their gene promoters^24^. VEGF is the key factor stimulating tumor angiogenesis. LHDA and GLUT1 are key proteins in the glycolysis cascade for tumor metabolism since cancer cells require high glycolytic rates and have become potential targets in cancer treatments by inhibiting cell proliferation and inducing apoptosis^40,41^. SOV inhibits the expression of HIF-1α and HIF-2α proteins and their nuclear translocation, leading to downregulation of GLUT1 and LHDA and promoting cell apoptosis^42^. Chronic exposure of sorafenib elevated the activity of Na^+^/K^+^-ATPase, contributing to the mechanisms for sorafenib resistance, while SOV reduces Na^+^/K^+^-ATPase activity, thus re-sensitizing SR-HCC cells. SOV upregulates the expression of cyclin B1 and increases the phosphorylation of cyclin B1 and CKD1, leading to cell cycle arrest at G2/M phases, thus inhibiting cell proliferation. But this activity of SOV seems to be independent of Na^+^/K^+^-ATPase. SOV treatment induces apoptosis by activating caspase cascades and PARP.

Hepatocarcinogenesis is far more complicated compared with some other forms of cancer since no “driver gene” has been identified for the development of HCC up to now, thus no drug targeting a single molecule has exhibited a significant beneficial effect in clinic. The current therapeutic strategies have to block a few molecules or pathways or their networks, resulting in that only drugs like sorafenib that inhibit multiple targets are able to show significant beneficial effects against HCC^2,3^. Unfortunately, sorafenib resistance greatly influences its beneficial effects in the treatment of HCC. Therefore, the present results may provide evidence for supporting further clinical investigation of SOV as a potential anti-cancer drug to enhance the efficacy of sorafenib to treat HCC, particularly sorafenib-resistant HCC.

## Materials and Methods

### Cells, antibodies, and reagents

Human HCC HepG2, Hep3B and SK-Hep-1 cells were obtained from the American Type Culture Collection, and Huh7 cells from Chinese Academy of Sciences Cell Bank (Shanghai, China). Cells were routinely cultured in Dulbecco’s Modified Eagle Medium (DMEM) (Gibco BRL, Grand Island, NY, USA) supplemented with 10% fetal bovine serum in an incubator at 37 °C. Hypoxic cells were induced by incubating them in a hypoxia chamber containing 1% O_2_, 5% CO_2_ and 95% N_2_ at 37 °C for 24 h. The SR-HCC cells, HepG2-SR and Huh7-SR, have previously been described^31,32^. They were stored in liquid nitrogen and resuscitation. Their ability of sorafenib resistance was further established and confirmed by incubating them with sorafenib at a starting concentration of 5 μM. Cells were continuously cultured with increasing concentrations of sorafenib by 1 μM per week for 1–2 months. The re-obtained SR-HCC cells were kept by culturing them in the presence of sorafenib. The detailed information for antibodies and reagents used in this study are described in Supplementary Information.

### ATPase activity assay

Cells were lysed in a protein lysate buffer (50 mM Tris pH 7.4, 100 μM EDTA, 0.25 M sucrose, 1% SDS, 1% NP40, 1 μg/ml leupeptin, 1 μg/ml pepstatin A and 100 μM phenyl methyl sulfonyl flouride) and homogenized. Debris was removed by centrifugation at 10,000 × g for 10 min at 4 °C, and protein concentrations were measured ATPase Activity Assay Kit (Sigma-Aldrich; malachite green assay, MAK113) following the manual provided by the manufacturer. Absorbance was read at 620 nm, and free phosphate was calculated.

### Measurement of intracellular potassium ion

The methods have been described previously^13^. Briefly, PBFI-AM (Potassium-binding benzofuran isophthalate-AM) (Invitrogen™ Molecular Probes™, Thermo Fisher Scientific), a cell permeant potassium indicator, was used for measuring intracellular K^+^ content. After cultured cells were washed with Hank’s balanced salt solution (HBSS), 5 μM PBFI and 10 μM F-127 were added to cells, which were incubated for 40 min at 5% CO_2_ and 37 °C in the dark. Cells were washed again with HBSS thrice, and then subjected to a fluorescent microscope (Leica DMIRB, Germany). Measurements were made by exciting PBFI at 340 nm and monitoring emission at 500 nm. The fluorescence intensity was assessed by using the NIH imaging software Image J.

### Animal experiments

The present study was approved by the Animal Ethics Committee of Harbin Medical University (permit SYXK20020009), and was in compliance with the Experimental Animal Regulations by the National Science and Technology Commission, China. The animal experimental protocol has previously been described in details previously^27,31,32^. Briefly, male BALB/c-nu/nu mice (aging 6–8 weeks) obtained from SLAC laboratory Animal Co., Ltd. (Shanghai, China) were maintained at the Animal Research Center of the First Affiliated Hospital of Harbin Medical University. Huh7-SR cells (5 × 10^6^) were subcutaneously injected into the flake of mice, which were orally administered with 10 mg/kg sorafenib daily and closely monitored. Any appearance of tumors was checked and recorded. Around 20 days after cell inoculation, the mice bearing subcutaneous tumors (∼100 mm^3^ in volume) were randomly assigned to four treatment groups. The vehicle solution contained Cremophor (Sigma-Aldrich), 95% ethanol and water in a ratio of 1:1:6. Sorafenib suspended in the vehicle solution was given to mice in the sorafenib and sorafenib plus SOV groups by gavage feeding at a dose of 30 mg/kg daily. SOV was administered into mice at the SOV and sorafenib plus SOV groups by intraperitoneal injection at a dose of 15 mg/kg daily. Mice in the control group received oral vehicle and intraperitoneal injection of PBS. Mice were monitored for record the size of tumors every 5 days and euthanized 20 days after treatments started.

### Other methodologies for analyzing cell proliferation, cell cycle, apoptosis and gene expression *in vitro* and *in vivo*

Please refer to Supplementary Information for detailed methods, which have also been described previously^21,27,31,32^.

### Statistical analysis

The data were expressed as mean values ± standard deviation (SD) from at least three independent experiments. Comparisons were made by using a one-way analysis of variance followed by a Dunnet’s t-test with the statistical software SPSS 18.0 for Windows (SPSS 224 Inc., IL, USA). *P* < 0.05 was considered significant.

### Data availability

The data generated or analyzed during this study are available from the corresponding author on reasonable request.

## Electronic supplementary material

Supplementary Information
