# Supplementary material for: Sodium orthovanadate overcomes sorafenib resistance of hepatocellular carcinoma cells by inhibiting Na+/K+-ATPase activity and hypoxia-inducible pathways
Source: Sci Rep. 2018 Jun 26;8:9706. doi: 10.1038/s41598-018-28010-y (PMC6018801; doi:10.1038/s41598-018-28010-y)
Supplement: Supplementary file 1 — Supplementary Information [file 41598_2018_28010_MOESM1_ESM.pdf]

Supplementary Information for:

Sodium orthovanadate overcomes sorafenib resistance of hepatocellular carcinoma cells by inhibiting Na^+/K^+-ATPase activity and hypoxia-inducible pathways

Wenjing Jiang[1], Guangxin Li[2], Weidong Li[1,3], Ping Wang[3,4], Peng Xiu[2], Xian Jiang[1], Bing Liu[1], Xueying Sun[1,3]*, Hongchi Jiang[1,]*

[1]Department of General Surgery, the First Affiliated Hospital of Harbin Medical University, Harbin 150001, China.

[2]Department of General Surgery, Qianfoshan Hospital Affliated to Shandong University, Jinan 250014, China

[3]The Hepatosplenic Surgery Center, the First Affiliated Hospital of Harbin Medical University, Harbin 150001, China.

[4]Department of Interventional Radiology, The Third Affiliated Hospital of Harbin Medical University, Harbin 150081, China.

*Correspondence and requests for materials should be addressed to Hongchi Jiang, (E-mail: jianghongchi@hrbmu.edu.cn) or Xueying Sun (Email: sunxueying@hrbmu.edu.cn or k.sun@auckland.ac.nz).

Contents

Supplementary Materials and Methods 3

Antibodies and reagents 3

Immunocytochemistry...4

Cell proliferation analysis..4

Assessment of cell cycle and apoptosis *in vitro*...4

Immunoblotting analysis..5

Quantitative reverse-transcription polymerase chain reaction (qRT-PCR)……….....6

Transfection of siRNA...6

Measurement of mitochondrial membrane potential..6

Immunohistochemistry and *In situ* Ki-67 proliferation index.................................7

In situ detection of apoptotic cells..7

References……………………………………………………………………..8

Genes examined, primers used for RT-PCR and PCR products (Table S1)..............9

Figure S1……………………………………………………………..............10

Figure S2……………………………………………………………..............11

Figure S3……………………………………………………………..............12

Figure S4……………………………………………………………..............13

Figure S5……………………………………………………………..............14

Figure S6……………………………………………………………..............15

Figure S7……………………………………………………………..............16

Figure S8……………………………………………………………..............17

Figure S9……………………………………………………………..............18

Full-length gels and blots of immunoblotting……..………………………………20

Supplementary Materials and Methods

Antibodies and reagents

An antibody (Ab) against Ki67 (ab15580) was from Abcam (Cambridge, MA, USA). Abs against phospho-cdc2 (Tyr161) (#9114), cyclin B1 (#4135) and phospho-cyclin B1 (Ser147) (#4131), PARP (#9542), caspase-9 (#9508) and caspase-3 (#9665) were purchased from Cell Signaling Technology (Danvers, USA). Abs against Na^+/K^+-ATPase α3 (sc-16052), CDK1 (sc-137035), HIF-1α (sc-13515), HIF-2α (sc-13596), ARNT (sc-17811), VEGF (sc-152), LDHA (sc-130327), GLUT1 (sc-377228) and β-actin (sc-130065), and a fluorescein isothiocyanate (FITC)-conjugated rat anti-mouse Ab (sc-516140) were from Santa Cruz Biotechnology (CA, USA). Sorafenib was from Jinan Trio Pharmatech Co., Ltd, and dissolved in dimethyl sulfoxide to make a stock solution of 10 mM. Sodium orthovanadate and ouabain octahydrate were from Sigma-Aldrich (Shanghai, China) and dissolved in phosphate-buffered saline (PBS). For animal experiments, sorafenib was suspended in the vehicle solution containing Cremophor (Sigma-Aldrich), 95% ethanol and water in a ratio of 1:1:6. The PI (propidium iodide)/Annexin V-FITC apoptosis detection kit was from BD Biosciences. The Cell Counting Kit-8 (CCK-8) kit was from Dojindo Molecular Technologies, Gaithersburg, MD, USA). Terminal deoxynucleotidyl transferase-mediated dUTP nick end labeling agent (TUNEL) was from Roche.

Immunocytochemistry

Cells were fixed with 4% paraformaldehyde, and permeabilized with 0.1% Triton X-100 in PBS. Cells were blocked in 10% normal serum for 30 min, and then incubated with a primary Ab against Na^+/K^+-ATPase α3 subunit for 90 min, followed by an FITC-conjugated rat anti-mouse Ab. Cells were visualized under confocal microscopy.

Cell proliferation analysis

Cells were seeded into a 96-well plate (3×10^3/well) in triplicate and cultured overnight. The culture medium was replaced with fresh FBS-free media containing vehicle or testing reagents at various concentrations. Cell viability was measured with a CCK-8 kit. Untreated cells served as controls. Cell viability (%) was calculated according to the formula: experimental OD value/control OD value ×100%.

Assessment of cell cycle and apoptosis *in vitro*

Cells were seeded at 5.0×10^5 cells/well in six-well plates and incubated with different reagents for 48 h, and then harvested and counted. A Cell Cycle kit (BD Biosciences, Beijing, China) was used to determine the percentages of cells at different phases of cell cycle by using flow cytometry with a Beckman Coulter Epics Altra II cytometer (Beckman Coulter, California, USA). Cells (1×10^5) were incubated in 110μl of binding buffer containing 5μl of Annexin V and 5μl of PI for 15 min at room temperature in dark, and then subjected to flow cytometry to measure the apoptosis rate (%) with the cytometer. Or cells were visualized under laser scanning confocal

microscopy.

Immunoblotting analysis

Cells or tumor tissues were homogenized in protein lysate buffer (50 mM Tris pH 7.4, 100 μM EDTA, 0.25 M sucrose, 1%SDS, 1% NP40, 1μg/ml leupeptin, 1μg/ml pepstatin A and 100 μM phenyl methyl sulfonyl flouride) and debris was removed by centrifugation at 10,000 × g for 10 min at 4°C. Protein concentrations were determined (Bio-Rad, Richmond, CA, USA). Lysates were resolved on sodium dodecyl sulfate-polyacrylamide (SDS-PAGE) gels, and then electrophoretically transferred to polyvinylidene difluoride (PVDF) membranes. The membranes were blocked in TBST (137 mM NaCl, 20 mM Tris HCl [pH 7.6], and 0.1% [v/v] Tween 20) containing 5% (w/v) nonfat dry milk at 37°C for 2 h, and then incubated overnight with primary Abs, and subsequently with alkaline phosphatase-conjugated secondary Abs for 2 h at room temperature in the dark. They were developed with 5-bromo-4-chloro-3-indolyl phosphate (BCIP)/ nitro blue tetrazolium (NBT) (Tiangen Biotech Co. Ltd., Beijing, China). The density of each band was measured using a densitometric analysis program (FR200, Shanghai, China). In preliminary experiments, serial dilutions of lysates (containing 2.5, 5, 10, 20, 40 or 80μg protein) were immunoblotted; band intensities were measured and plotted against protein amounts to generate a standard curve, and the amount of protein for each blot was determined.

Quantitative reverse-transcription polymerase chain reaction (qRT-PCR)

Methods have been described in details previously [1,2]. Briefly, total RNA was extracted from cells, and cDNA synthesized. The reaction mixtures for qRT-PCR were prepared with the primers as shown in Table S1. The PCR products were analyzed by MX3000P Real-time PCR systems (Stratagen, USA). Experiments were performed in triplicate, and data were calculated by ΔΔCt methods.

Transfection of siRNA

The methods have been described previously[3,4]. A siRNA targeting human Na+/K+-ATPase α3 subunit (5'-ACGACAACCGAUACCUGCUGGUGAU-3' and 5'-AUCACCAGCAGGUAUCGGUUGUCGU-3') and a control nonspecific scrambled siRNA (5'-UUCUCCGAACGUGUCACGU-3' and 5'-ACGUGACACGUUCGGAGAA-3') were purchased from GenePharma (Shanghai, China). Cells were grown to 60-70% confluence, and incubated with siRNAs at a final concentration of 0.1 μM by using Lipofectamine™ 2000 (Invitrogen, Beijing, China) in a serum-free medium for 48 h and then subjected to the assays.

Measurement of mitochondrial membrane potential

A lipophilic cationic dye, JC-1, was used to measure changes in mitochondrial membrane potential (ΔΨM). Cells were incubated with 10 μg/ml of JC-1 for 20 min at 37°C, washed three times and then resuspended in PBS at 1×10^6 cells/ml. Cells were analyzed by flow cytometry at an excitation wavelength of 514 nm. Data were

collected at the emission wavelength of 529 nm (green fluorescence) of the JC-1 monomer and at 585 nm (red fluorescence) for JC-1 aggregates. The ratio of red/green fluorescence intensities was recorded, and the relative ΔΨM was calculated.

Caspase-3 activity assay

Cells were lysed and 100 μg of lysates were incubated with caspase-3 colorimetric DEVD-pNA substrate at 37°C. The resulting colorimetric product was measured with a microplate spectrophotometer at 402 nm according to the instructions, supplied in the caspase-3 activity kit (ab39401, Abcam, Shanghai, China). Caspase-3 activity was calculated as a fold change in comparison to cells without treatment. All conditions were run in duplicate and three independent experiments were performed.

Immunohistochemistry and In situ Ki-67 proliferation index

Formalin fixed tumor specimens were transferred to 70% ethanol and subsequently paraffin-embedded and sectioned. Tumor sections were rinsed with PBS, blocked with 3% BSA for 2 h, and incubated with Abs against Ki-67, p-Akt, p-Met, or cleaved caspase-3 at 4°C overnight. They were subsequently incubated for 30 min with the appropriate secondary Ab using the Ultra-Sensitive TMS-P kit (Zhongshan Co., Beijing, China), and immunoreactivity developed with Sigma FAST DAB (3,3'-diaminobenzidine tetrahydrochloride) and $CoCl_2$ enhancer tablets (Sigma-Aldrich, Shanghai, China). Sections were counterstained with hematoxylin, mounted, and examined by microscopy. The Ki-67 positive cells were counted in 10

randomly selected × 400 high-power fields under microscopy. The Ki-67 proliferation index was calculated according to the following formula: the number of Ki-67 positive cells/ the total cell count × 100%.

In situ detection of apoptotic cells

The above tumor sections were stained with the TUNEL (Terminal deoxynucleotidyl transferase dUTP nick end labeling) (Roche, Shanghai, China). The TUNEL positive cells were counted in 20 randomly selected × 200 high-power fields under microscopy. The apoptosis index was calculated according to the following formula: the number of apoptotic cells × /total number of nucleated cells × 100%.

References

1 He, C. *et al.* MiR-21 mediates sorafenib resistance of hepatocellular carcinoma cells by inhibiting autophagy via the PTEN/Akt pathway. *Oncotarget* **6**, 28867-28881, doi:10.18632/oncotarget.4814 (2015).

2 Zhai, B. *et al.* Inhibition of Akt reverses the acquired resistance to sorafenib by switching protective autophagy to autophagic cell death in hepatocellular carcinoma. *Molecular cancer therapeutics* **13**, 1589-1598, doi:10.1158/1535-7163.mct-13-1043 (2014).

3 Han, P. *et al.* Dual inhibition of Akt and c-Met as a second-line therapy following acquired resistance to sorafenib in hepatocellular carcinoma cells. *Molecular oncology* **11**, 320-334, doi:10.1002/1878-0261.12039 (2017).

4 Chen, D., Song, M., Mohamad, O. & Yu, S. P. Inhibition of Na+/K+-ATPase induces hybrid cell death and enhanced sensitivity to chemotherapy in human glioblastoma cells. *BMC cancer* **14**, 716, doi:10.1186/1471-2407-14-716 (2014).

Table S1. Genes examined, primers used for RT-PCR and PCR products

Protein Name	Gene Name	GenBank No.	Primer	PCR Product
α1	ATP1A1	NM_001160234.1	Forward: 5′-GAAAGAAGTTTCTATGGATG-3′<br>Reverse: 5′-ATG ATTACAACGGCTGATAG-3′	318bp (nt 280-597)
α2	ATP1A2	NM_000702.3	Forward: 5′-AGAGAATGGGGGCGGCAAG-3′<br>Reverse: 5′-TGGTTCATCCTCCATGGCAGCC-3′	322bp (nt 180-501)
α3	ATP1A3	NM_152296.4	Forward: 5′-CCTCACTCAGAACCGCATGAC-3′<br>Reverse: 5′-TTCATCACCAGCAGGTATCGG-3′	384bp (nt1310-1693)
α4	ATP1A4	NM_144699.3	Forward: 5′-TATTTCAATGAGGAGCCTAC-3′<br>Reverse: 5′-CTGTGGAGTCTCCCGTAGC-3′	419bp (nt 816-1279)
β1	ATP1B1	NM_001677.3	Forward: 5′-ACTGAAATTTCCTTTCGTCCTAAT-3′<br>Reverse: 5′-ATCACTGGGTAAGTCTCCA-3′	350bp (nt377-726)
β2	ATP1B2	NM_001678.4	Forward: 5′- CTTGATGTCATTGTCAATGTCAGT-3′<br>Reverse: 5′-TCGATGTTGCCGTTGGCGGGGAAC-3′	401bp (nt900-1300)
HIF-1α	HIF1A	NM_181054.2	Forward: 5′-ATCCATGTGACCATGAGGAAATG-3′<br>Reverse: 5′-CTCGGCTAGTTAGGGTACACTT-3′	126bp (nt 814-939)
HIF-2α	HIF2A	NM_001430.4	Forward: 5′-GTGCCATGACAAACATCTTCCAG-3′<br>Reverse: 5′-CTCGGGCTCTGTCTTCTTGCT-3′	107bp (nt 2204-2310)
VEGF	VEGF	AF022375	Forward: 5′-GTCCCAGGCTGCACCCATG-3′<br>Reverse: 5′-AGGAAGCTCATCTCTCCTA-3′	158 bp (nt 522-679)
LDHA	LDHA	X02152	Forward: 5′-GCTGGTCATTATCACGGCTG-3′<br>Reverse: 5′-AGCAACTTGCAGTTCGGGCTG-3′	128 bp (nt 361-488)
GLUT1	GLUT1	K03195	Forward: GAGTTCTACAACCAGACATGG	167bp (nt 302-468)
GAPDH	GAPDH	NM_002046.5	Forward: 5′- CACCCATGGCAAATTCCATGGCA-3′<br>Reverse: 5′-TCTAGACGGCAGGTCA GGTCCACC-3′	735bp (nt341-1075)

Notes: Protein names (α1, α2, α3, α4, β1 and β2) refer to subunits of Na^{+}/K^{+}-ATPase. Abbreviations: HIF, hypoxia-inducible factor; VEGF, vascular endothelial growth factor; LDHA, lactate dehydrogenase-A; GLUT1, glucose transporter 1; GAPDH, glyceraldehyde 3-phosphate dehydrogenase.

Figure S1

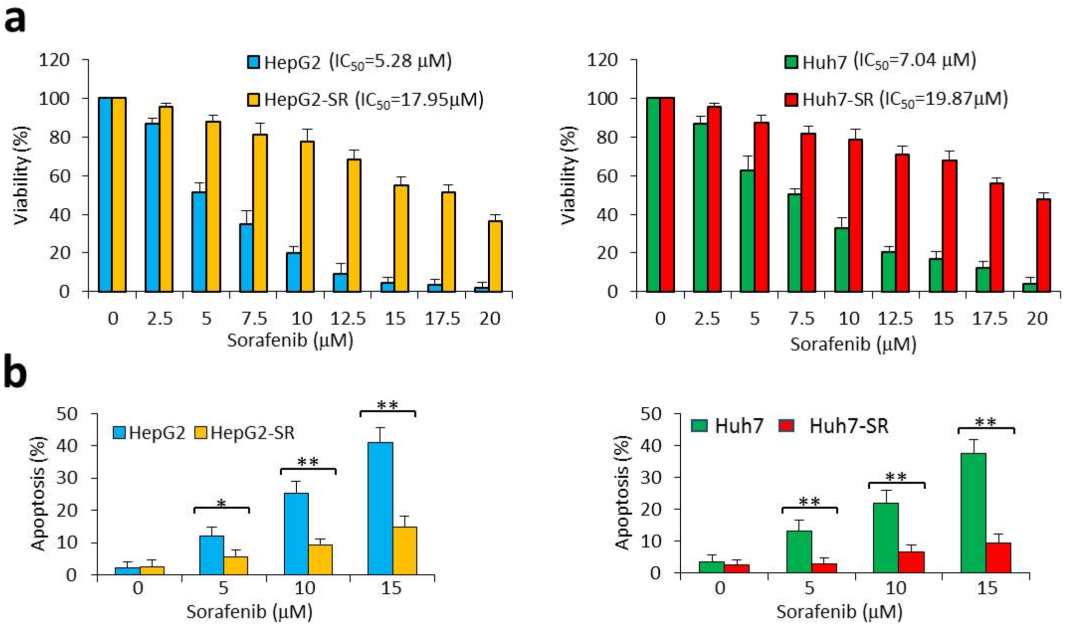

Figure S1. Sorafenib-resistant HCC cells are refractory to sorafenib-induced proliferation inhibition and apoptosis. (**a**) Sorafenib-resistant cells, HepG2-SR and Huh7-SR, and their parental HepG2 and Huh7 cells were incubated for 48 h with sorafenib at various concentrations as indicated. Cell viability (%) was compared with the respective untreated cells. The values of IC_{50} for each cell type were calculated. (**b**) Cells were incubated for 48 h with 0, 5, 10 or 15μM of sorafenib, and then analyzed to measure apoptosis rate (%). The density of each immunoblotting band was normalized to β-actin. “*” indicates P<0.05, and “**”, P<0.001.

Figure S2

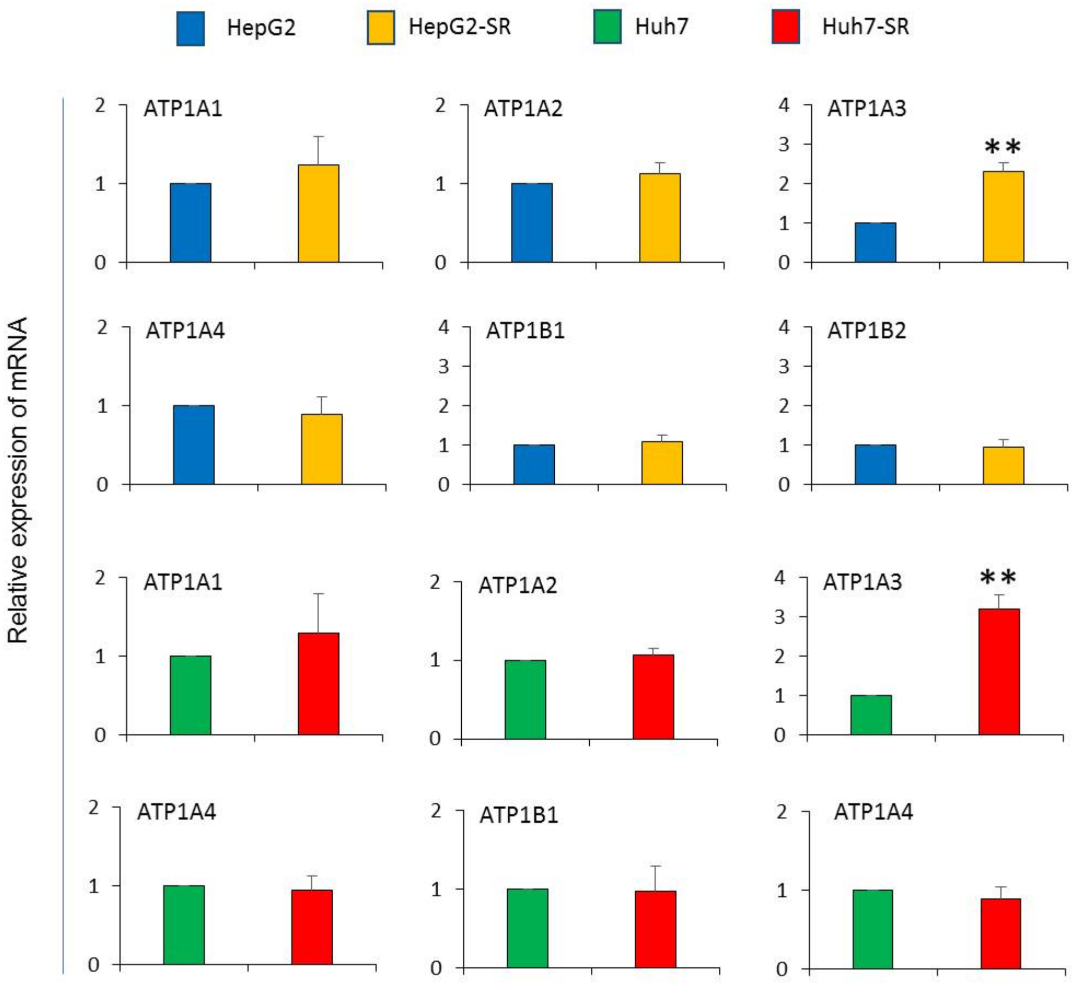

Figure S2. Expression of mRNA encoding Na^+/K^+-ATPase subunits in HCC cells. HepG2, HepG2-SR, Huh7 and Huh7-SR cells were subjected to qRT-PCR with primers as shown in Table S1. The expression level from parental cells was defined 1. “**” (P<0.001) indicates a significant difference from respective parental cells.

Figure S3

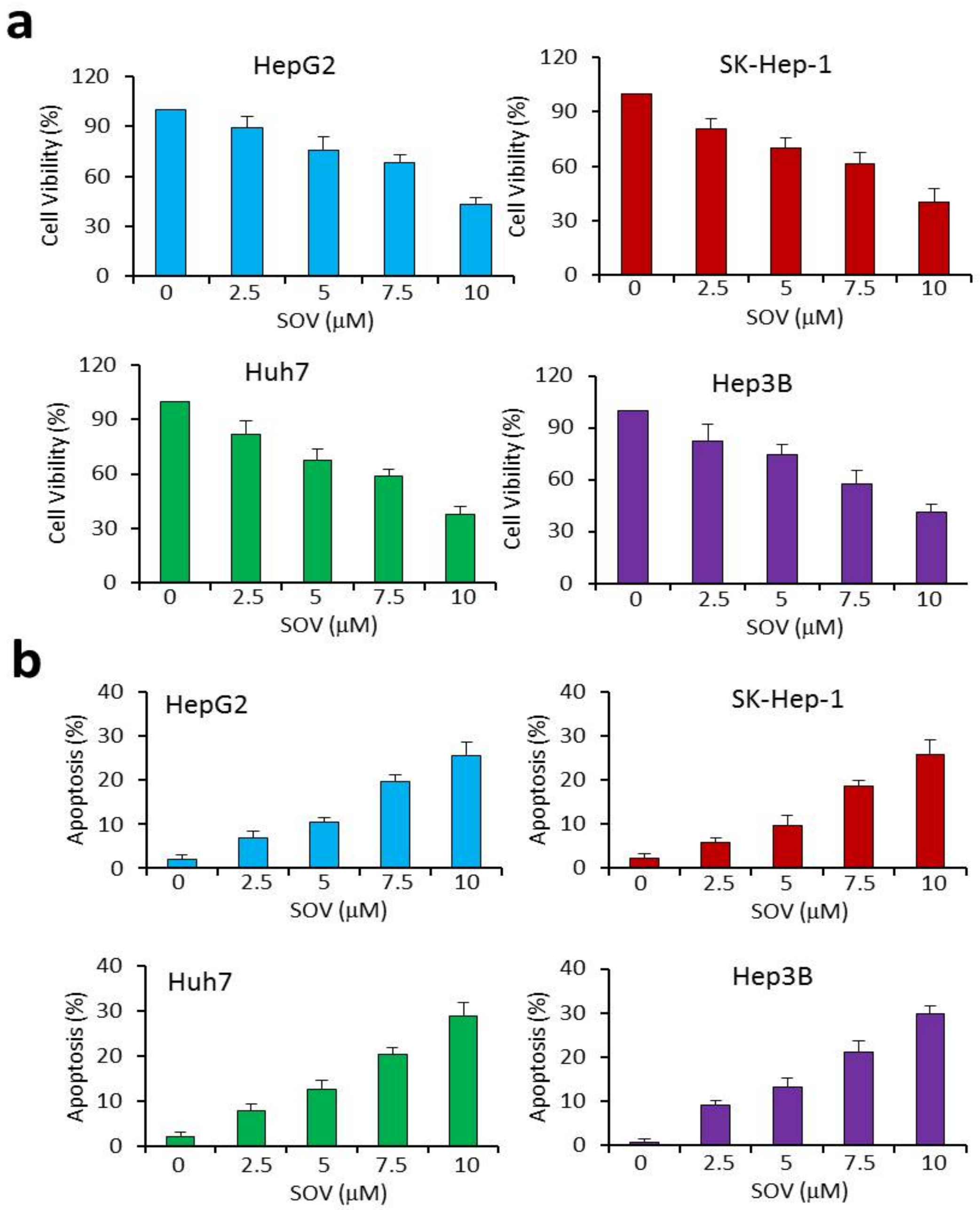

Figure S3. SOV inhibits the proliferation and induces the apoptosis of HCC cells. HepG2, HepG2-SR, Huh7 and Huh7-SR cells were incubated for 48 h with SOV at various concentrations. (**a**) Cell viability (%) was compared with the respective untreated cells. (**b**) The apoptosis rate (%) was measured.

Figure S4

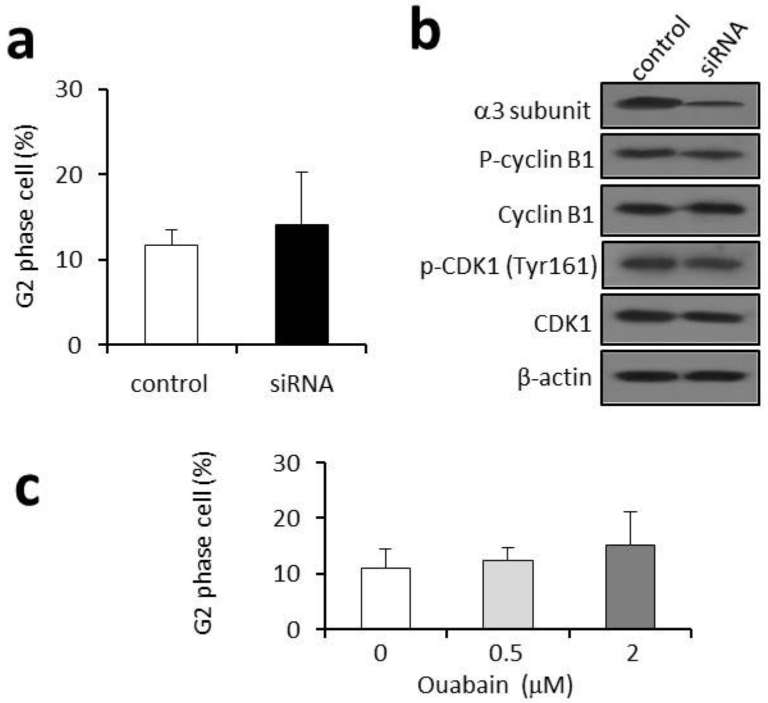

Figure S4. Inhibition of Na^{+}/K^{+}-ATPase does not induce cell cycle arrest. Huh7-SR cells were transfected with siRNA targeting Na^{+}/K^{+}-ATPase α3 subunit for 24 h. (**a**) Cells were cytometrically analyzed for cell cycle distribution. The percentages of cells arrested at G2/M phases were plotted. (**b**) Cells were subjected to immunoblotting. The density of each band was normalized to β-actin. (**c**) Cells were incubated for 24 with ouabain at concentrations of 0, 0.5 or 2 μM, and then subjected to cytometry for determining cell cycle distribution. The percentages of cells arrested at G2/M phases were plotted.

Figure S5

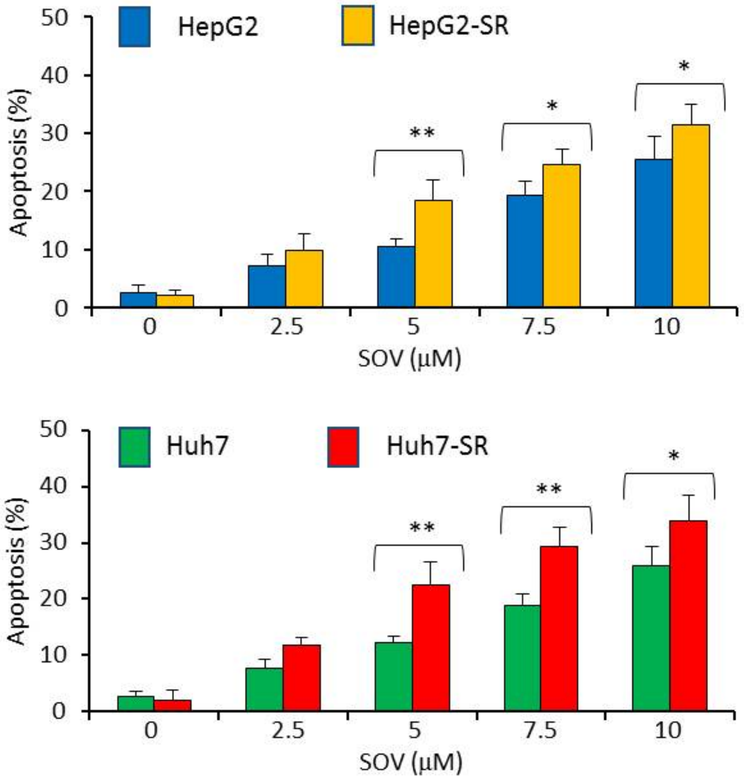

Figure S5. SOV induces apoptosis of HCC cells. HepG2, HepG2-SR, Huh7 and Huh7-SR cells were incubated for 24 h with various concentrations of SOV, and then subjected to apoptosis assays. “*” indicates P<0.05, and “**”, P<0.001.

Figure S6

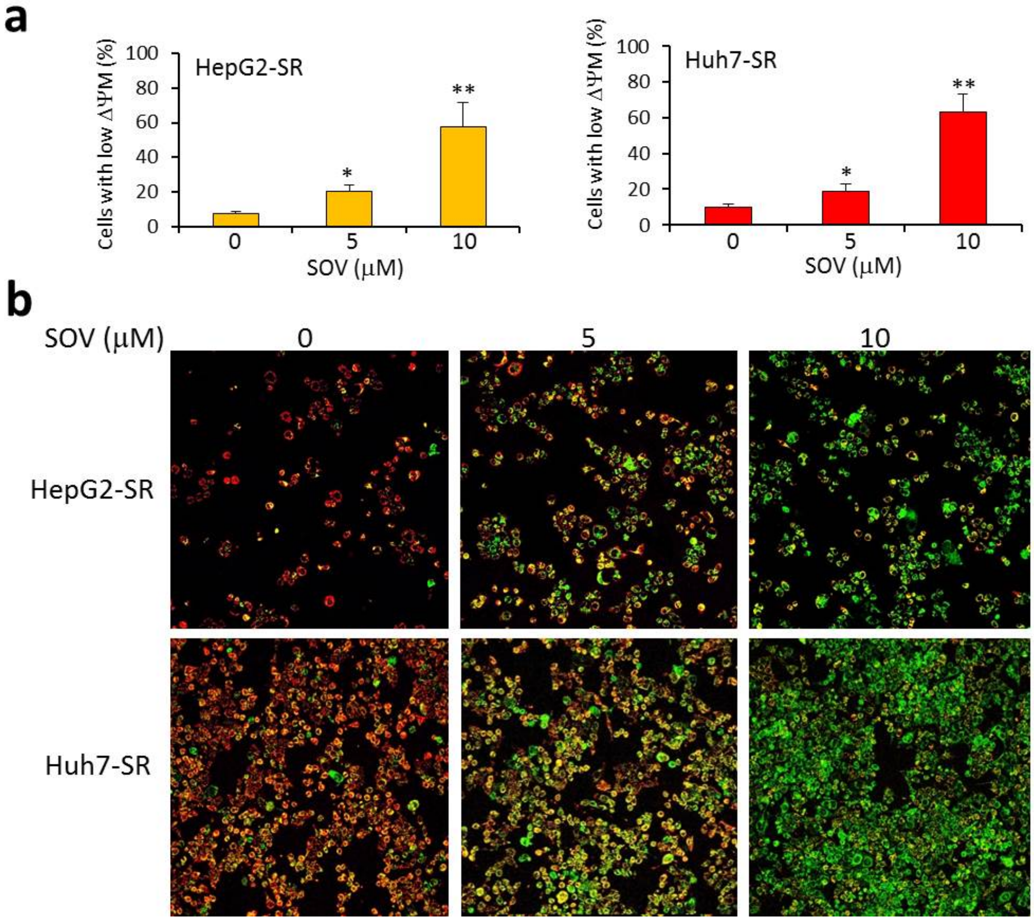

Figure S6. SOV reduces mitochondrial membrane potential (ΔΨM) of sorafenib-resistant HCC cells. HepG2-SR and Huhb7-SR cells were incubated with SOV (0, 5, 10 μM) for 24 h, and then stained with JC-1. (**a**) Cells with low ΔΨM (%) were plotted. (**b**) Representative images were from the above cells examined by fluorescence microscopy for detecting ΔΨM. "*" ($P<0.05$) and "**" ($P<0.001$) vs. untreated cells.

Figure S7

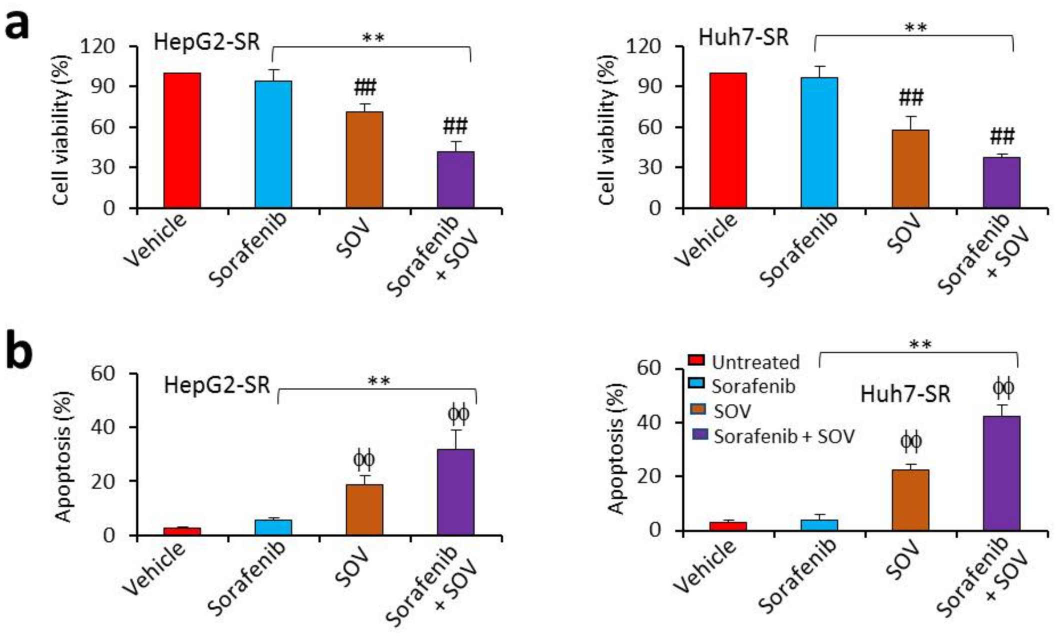

Figure S7. SOV enhances the effects of sorafenib against sorafenib-resistant HCC cells. HepG2-SR and Huh7-SR cells were incubated for 48 h with sorafenib (2.5µM), SOV (5µM) or their combination. Cells incubated with vehicle served as controls. (**a**) Cell viability (%) and (**b**) apoptosis rate (%) were measured. "*" indicates P<0.05, and "**" P<0.001. "#" (P<0.05) and "##" (P<0.001) indicate a significant reduction, and "ϕ" (P<0.05) and "ϕϕ" (0.001), a significant increase, from vehicle-treated cells.

Figure S8

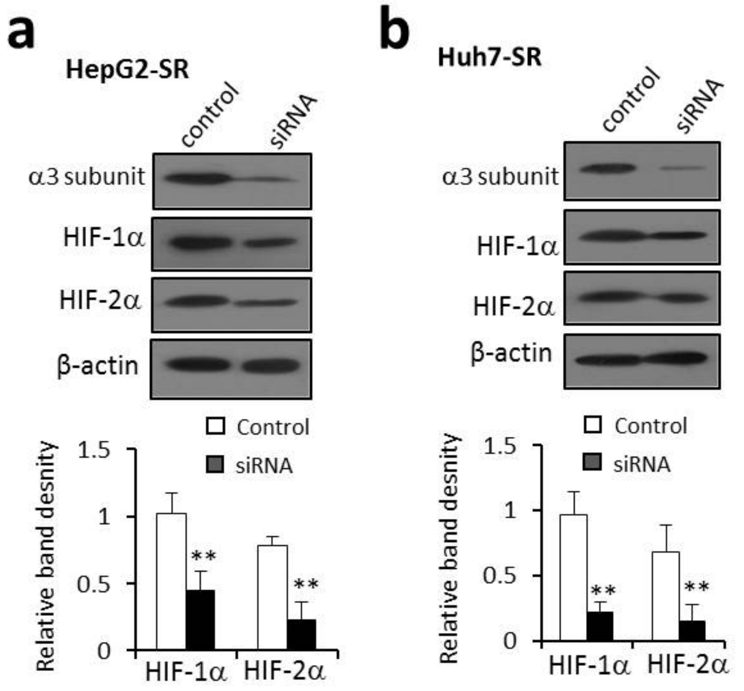

Figure S8. Depletion of Na^+/K^+-ATPase α3 subunit downregulates the expression of HIF-1α and HIF-2α proteins. HepG2-SR (**a**) and Huh7-SR (**b**) cells were transfected with control siRNA or siRNA targeting Na^+/K^+-ATPase α3 subunit for 24 h, and subjected to immunoblotting. The density of each immunoblotting band was normalized to β-actin. "**" ($P<0.001$) indicates significant difference from control siRNA-transfected cells.

Figure S9

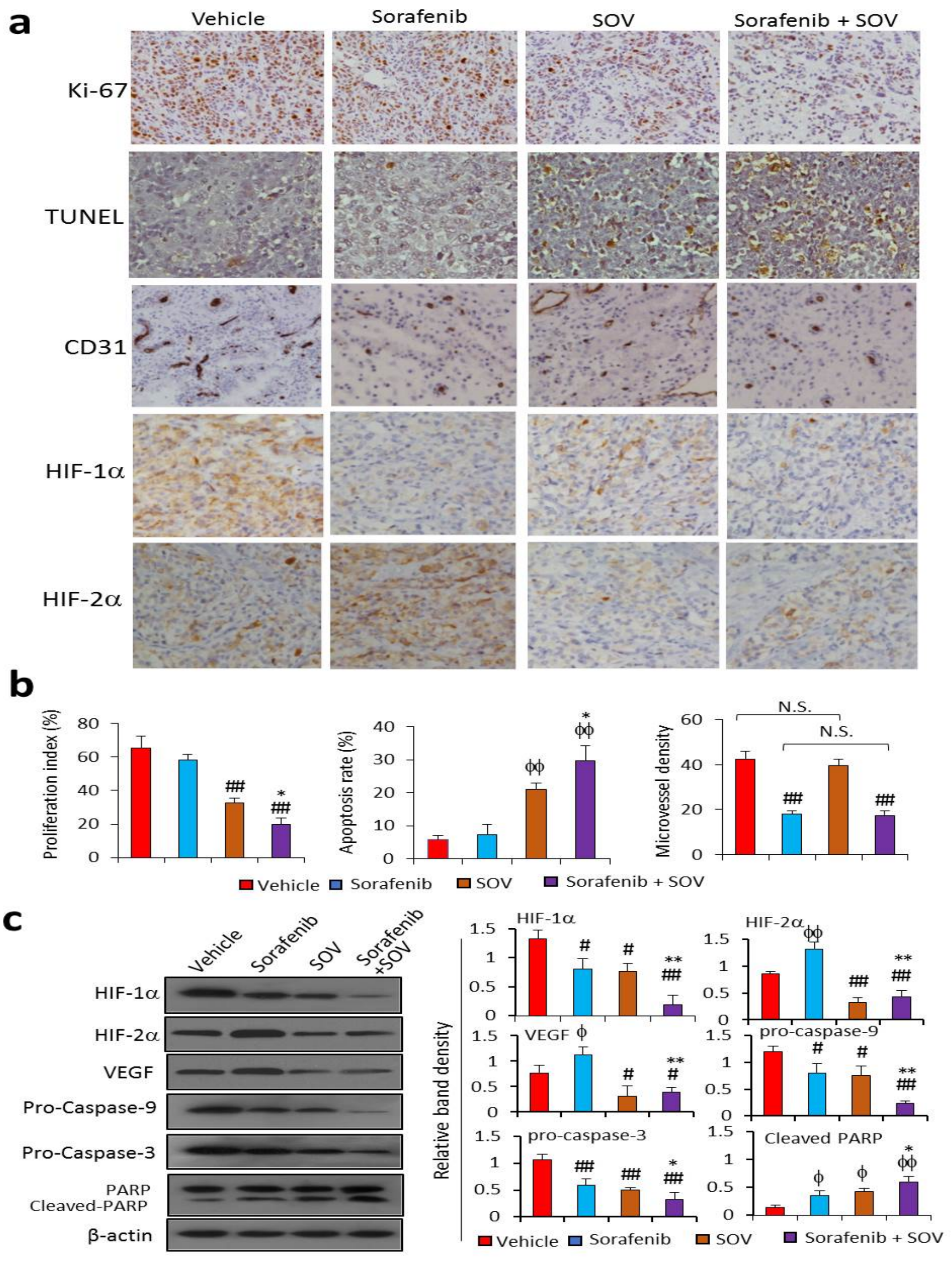

Figure S9. Cell proliferation, apoptosis, angiogenesis and gene expression in vivo. (**a**) Representative images of tumor sections taken from Figure 7 were immunostained with an anti-Ki67 Ab, TUNEL, anti-CD31 Ab, anti-HIF-1α Ab, or anti-HIF-2α Ab. (**b**) Proliferation index (%), apoptosis index (%) and microvessel density were quantified. (**c**) Tissue homogenates from tumors taken from Figure 7 were immunoblotted. The density of each immunoblotting band was normalized to β-actin. "#" ($P<0.05$) and "##" ($P<0.001$) indicate a significant reduction, and "ϕ" ($P<0.05$) and "ϕϕ" (0.001), a significant increase, from vehicle-treated tumors. "*" ($P<0.05$) and "**" ($P<0.001$) vs. sorafenib alone-treated tumors.

Full-length gels and blots of immunoblotting

Figure 1. b

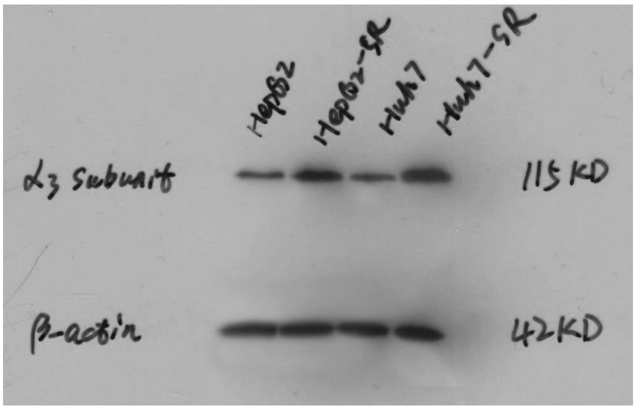

Figure 1. d

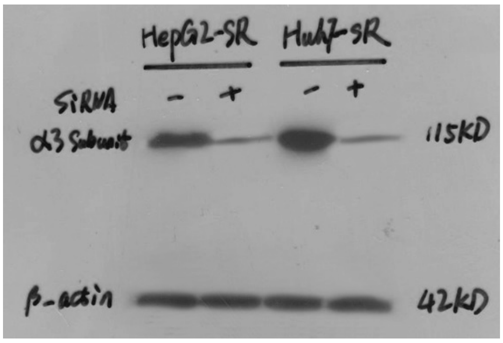

Figure. 2e

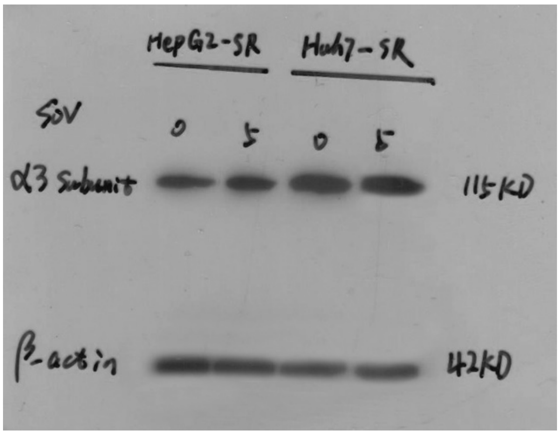

Figure 3d

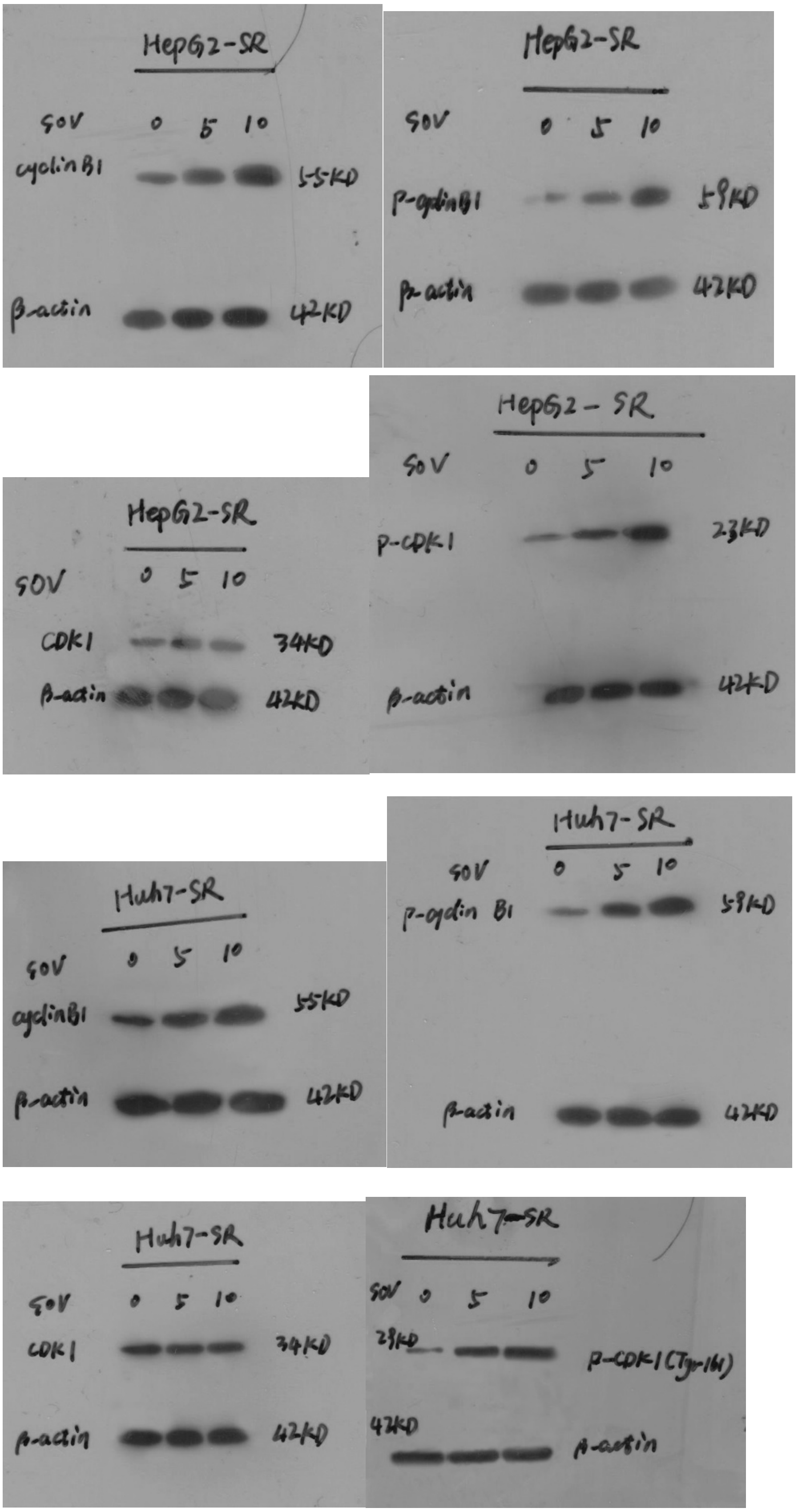
HepG2-SR
SOV 0 5 10
cyclin B1 55KD
β-actin 42KD
HepG2-SR
SOV 0 5 10
P-cyclin B1 59KD
β-actin 42KD
HepG2-SR
SOV 0 5 10
CDK1 34KD
β-actin 42KD
HepG2-SR
SOV 0 5 10
P-CDK1 23KD
β-actin 42KD
Huh7-SR
SOV 0 5 10
cyclin B1 55KD
β-actin 42KD
Huh7-SR
SOV 0 5 10
P-cyclin B1 59KD
β-actin 42KD
Huh7-SR
SOV 0 5 10
CDK1 34KD
β-actin 42KD
Huh7-SR
SOV 0 5 10
23KD P-CDK1 (Tyr-161)
42KD β-actin

Figure. 4c

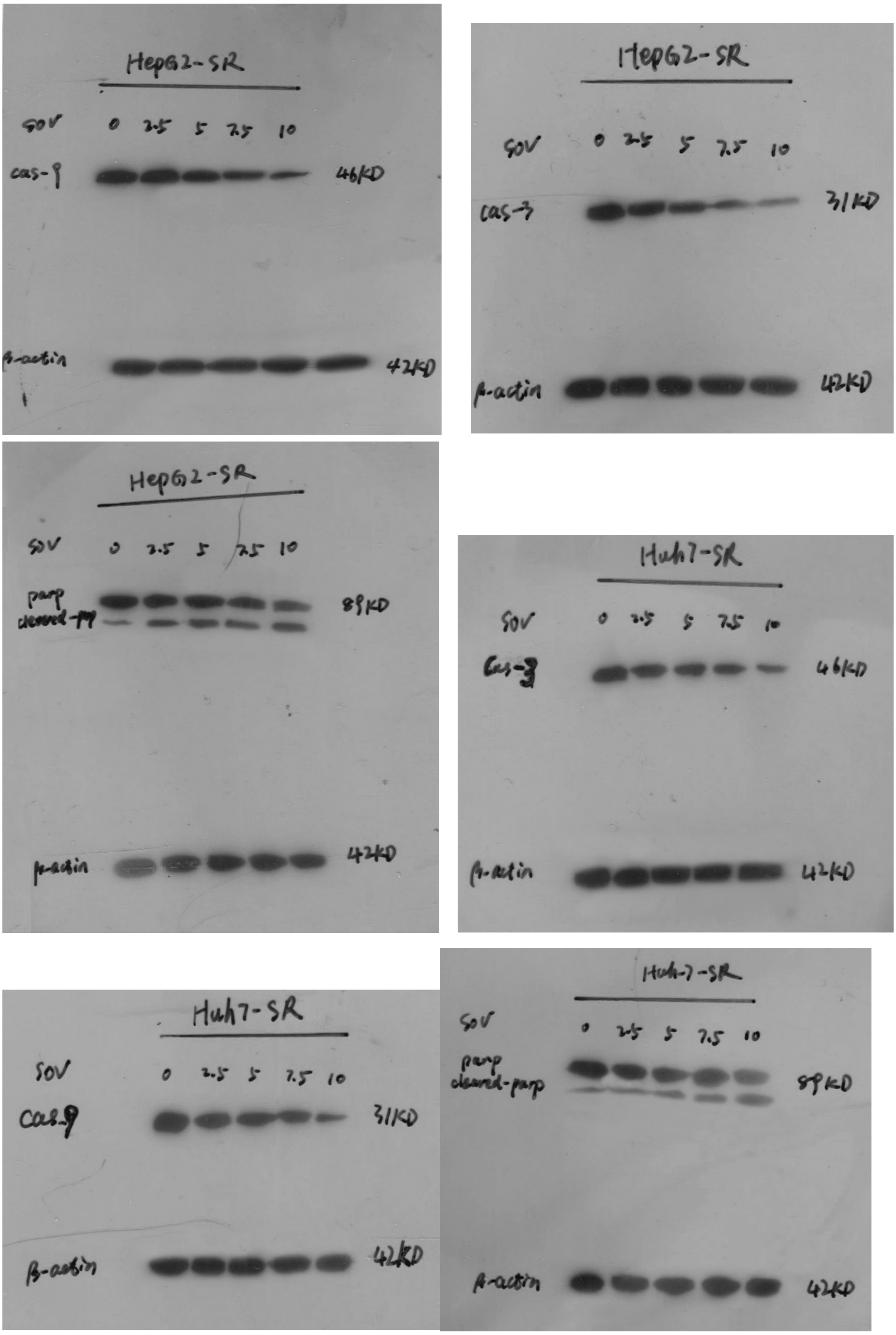
HepG2-SR
SOV 0 2.5 5 7.5 10
cas-9 46KD
β-actin 42KD
HepG2-SR
SOV 0 2.5 5 7.5 10
cas-3 31KD
β-actin 42KD
HepG2-SR
SOV 0 2.5 5 7.5 10
parp
cleaved-parp
89KD
β-actin 42KD
Huh7-SR
SOV 0 2.5 5 7.5 10
Cas-9 46KD
β-actin 42KD
Huh7-SR
SOV 0 2.5 5 7.5 10
Cas-9 31KD
β-actin 42KD
Huh-7-SR
SOV 0 2.5 5 7.5 10
parp
cleaved-parp
89KD
β-actin 42KD

Figure. 5a

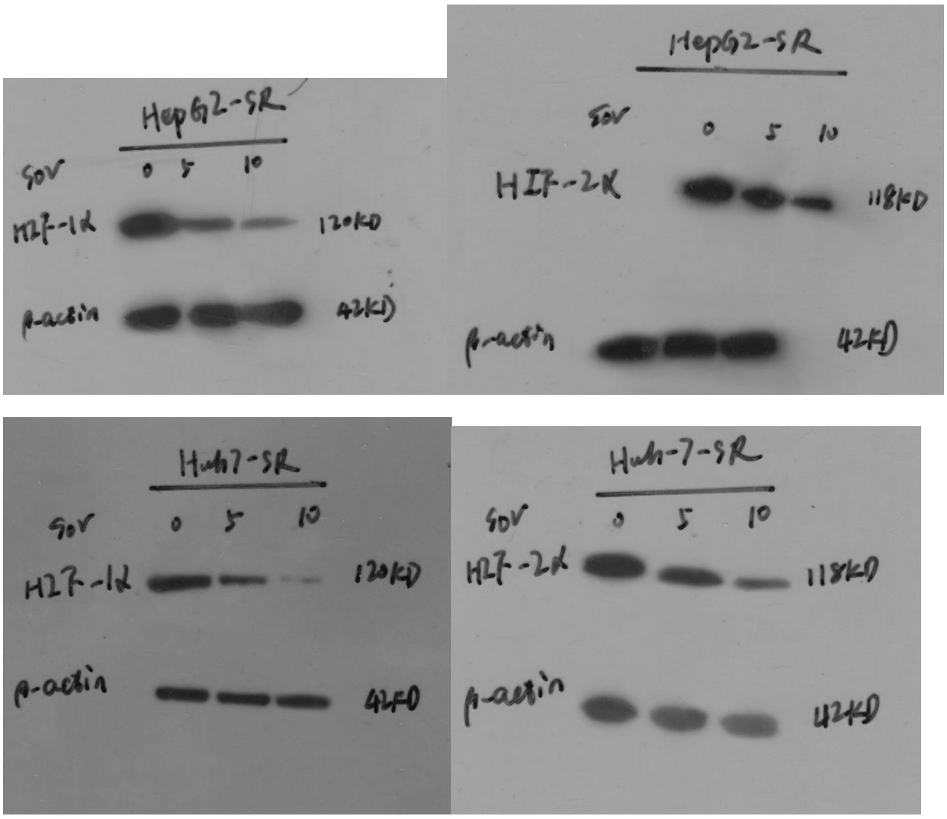

Figure. 5b

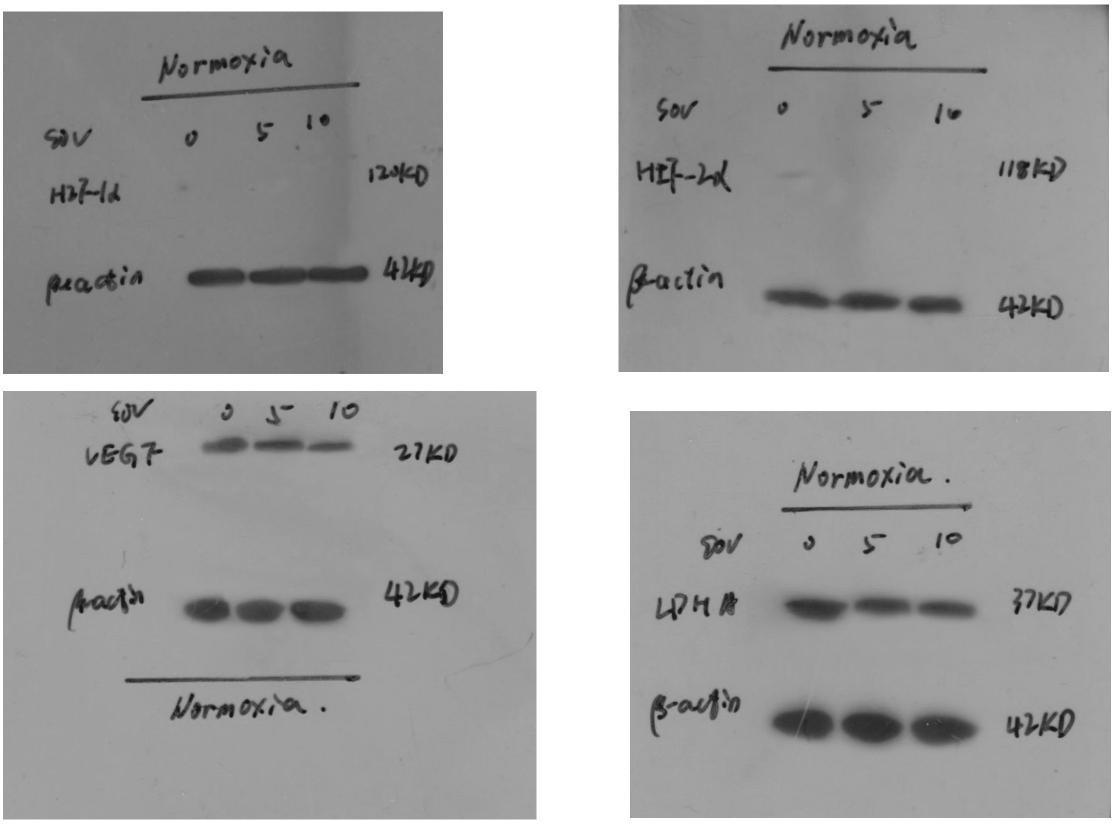

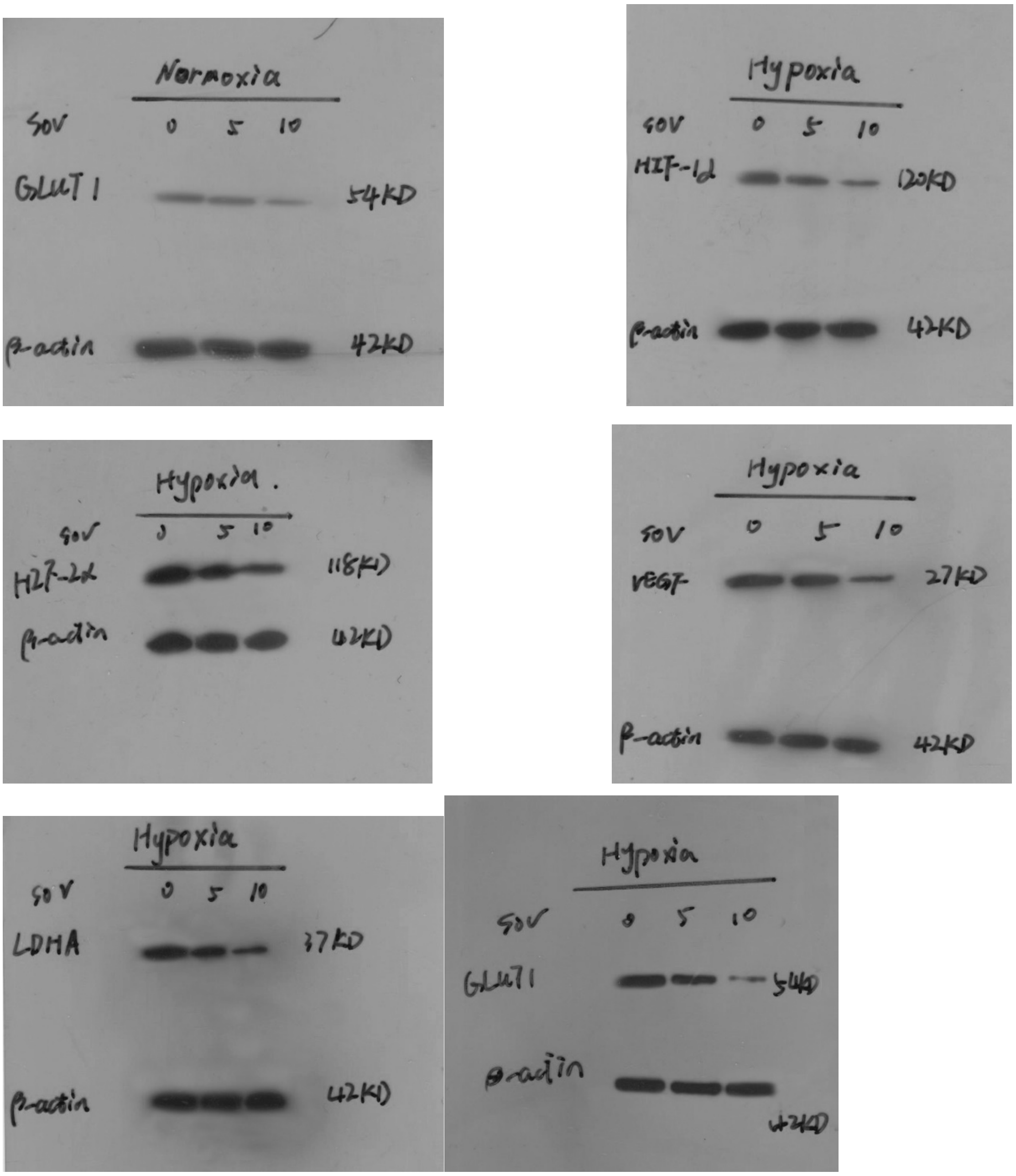
Normoxia
SOV 0 5 10
GLUT1 54KD
β-actin 42KD
Hypoxia
SOV 0 5 10
HIF-1α 120KD
β-actin 42KD
Hypoxia
SOV 0 5 10
HIF-2α 118KD
β-actin 42KD
Hypoxia
SOV 0 5 10
VEGF 27KD
β-actin 42KD
Hypoxia
SOV 0 5 10
LDHA 37KD
β-actin 42KD
Hypoxia
SOV 0 5 10
GLUT1 54KD
β-actin
42KD

Figure. 6c

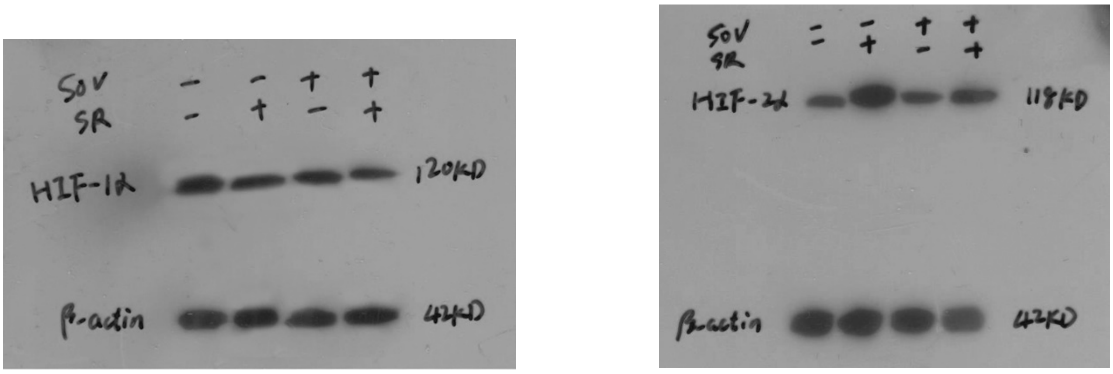
SOV - - + +
SR - + - +
HIF-1α 120KD
β-actin 42KD
SOV - - + +
SR - + - +
HIF-2α 118KD
β-actin 42KD

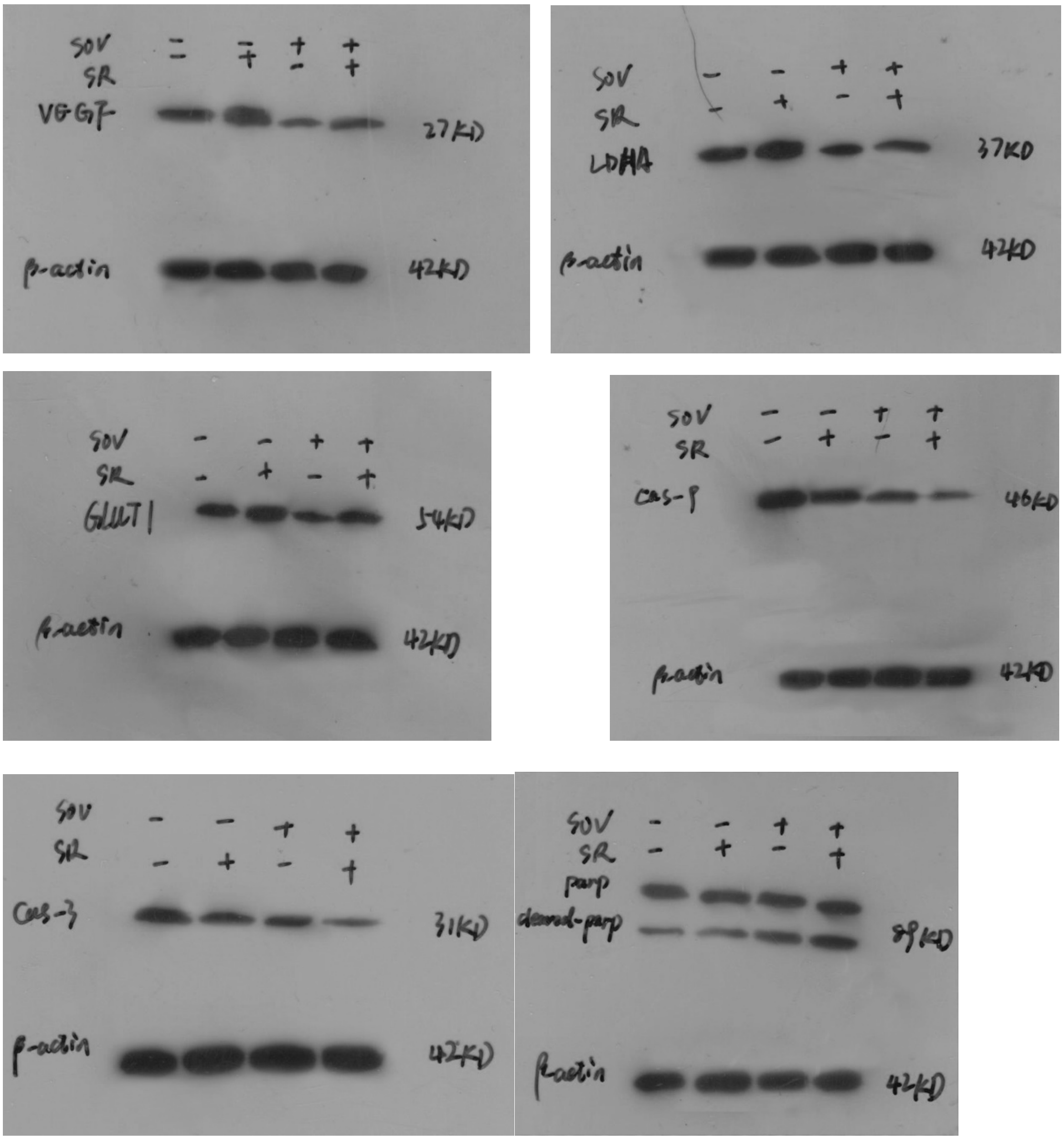
SOV
SR
VEGF
27KD
β-actin
42KD
LDHA
37KD
GLUT1
54KD
Cas-9
46KD
Cas-3
31KD
parp
cleaved-parp
89KD

Figure S4b

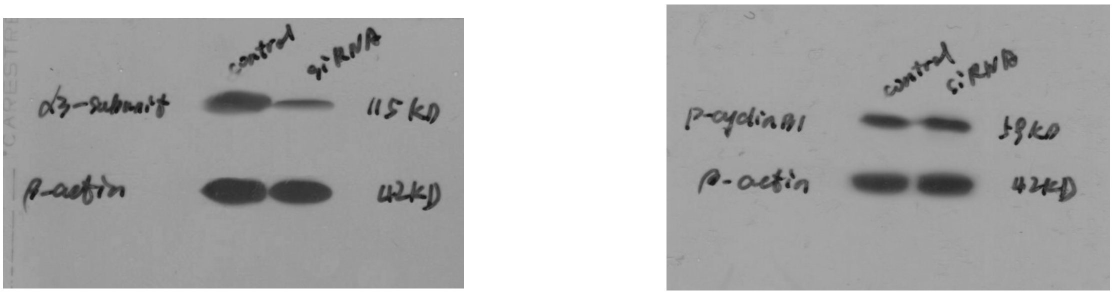
control
siRNA
115 KD
β-actin
42KD

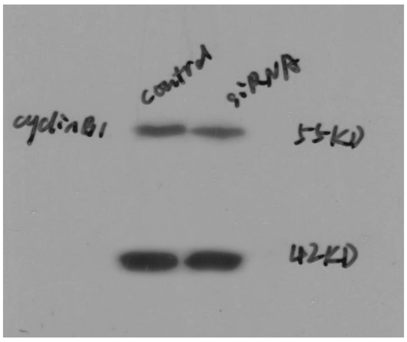
control
siRNA
cyclinB1
55KD
42KD

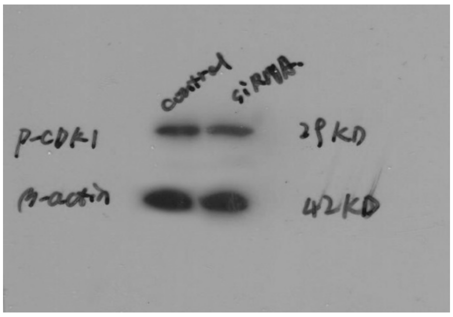
control
siRNA
p-CDK1
29KD
β-actin
42KD

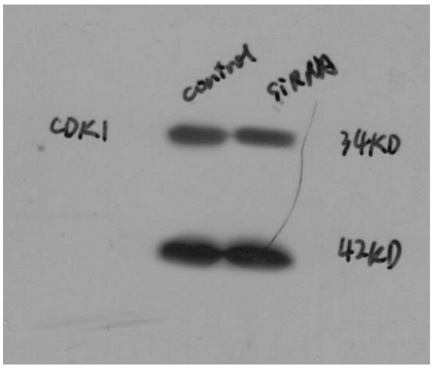
control
siRNA
CDK1
34KD
42KD

Figure S8a

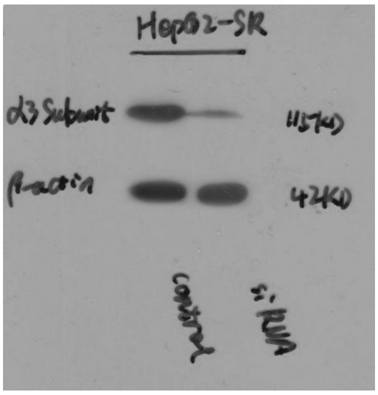
HepG2-SR
β-actin
42KD
control
siRNA

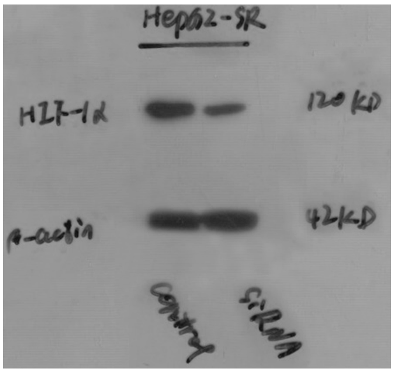
HepG2-SR
120 KD
β-actin
42KD
control
siRNA

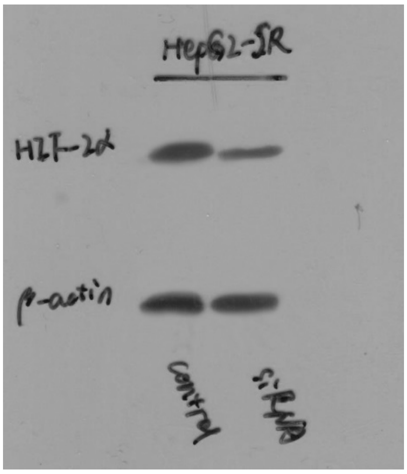
HepG2-SR
β-actin
control
siRNA

Figure S8b

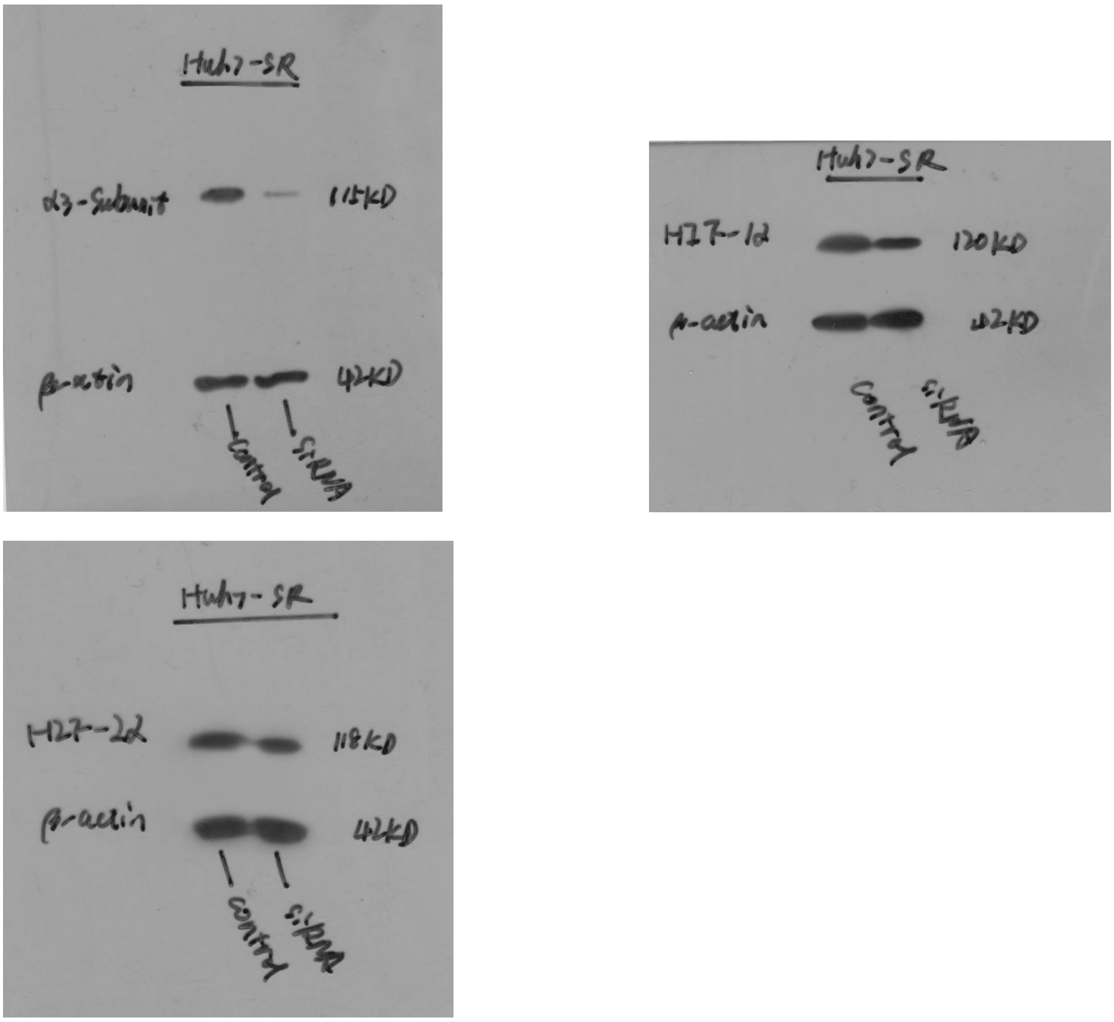

Figure S9c

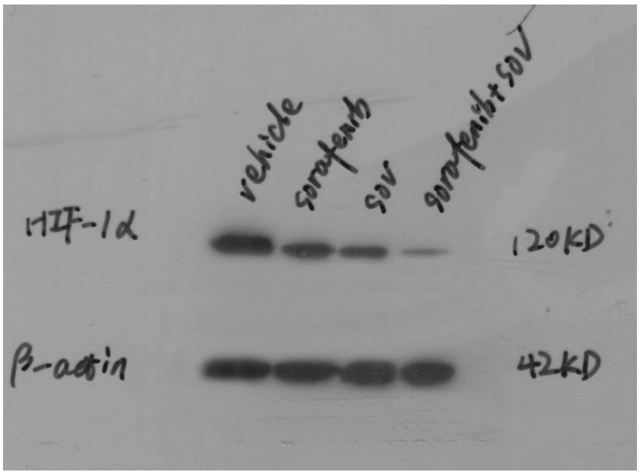

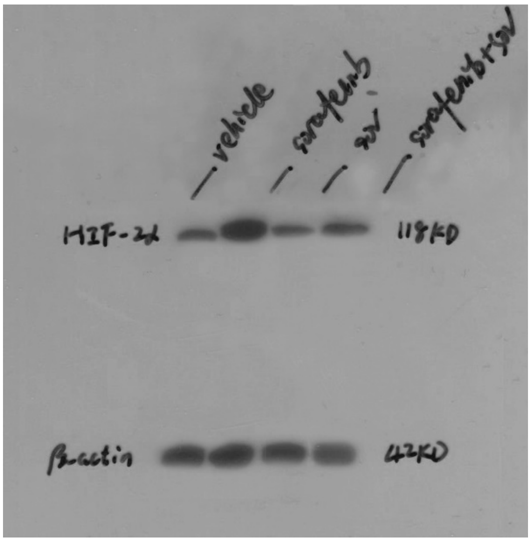
vehicle
sorafenib
SOV
sorafenib+SOV
HIF-2α
118KD
β-actin
42KD

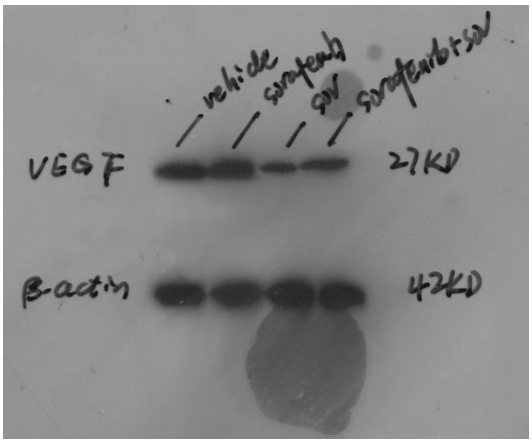
vehicle
sorafenib
SOV
sorafenib+SOV
VEGF
27KD
β-actin
42KD

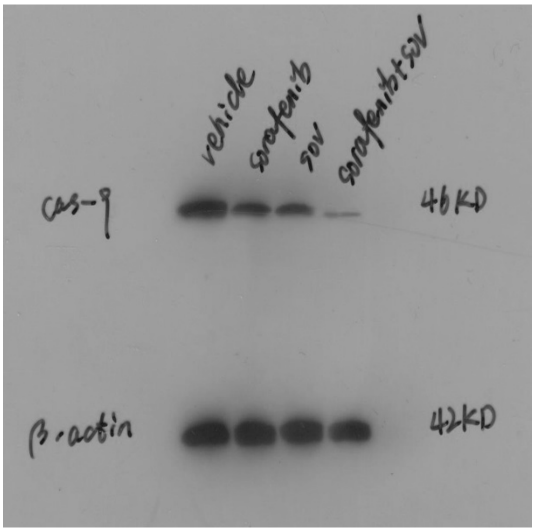
vehicle
sorafenib
SOV
sorafenib+SOV
cas-9
46KD
β-actin
42KD

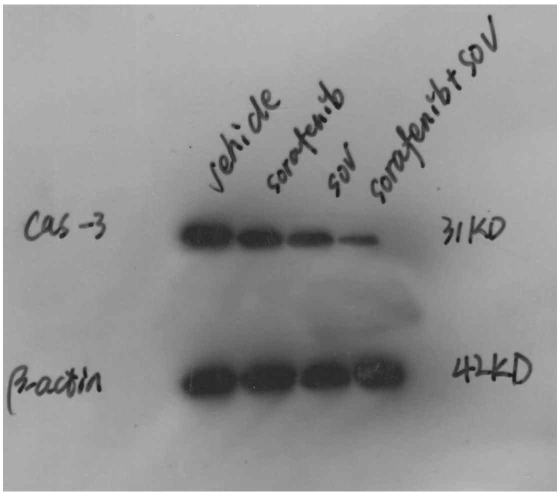
vehicle
sorafenib
SOV
sorafenib+SOV
Cas-3
31KD
β-actin
42KD

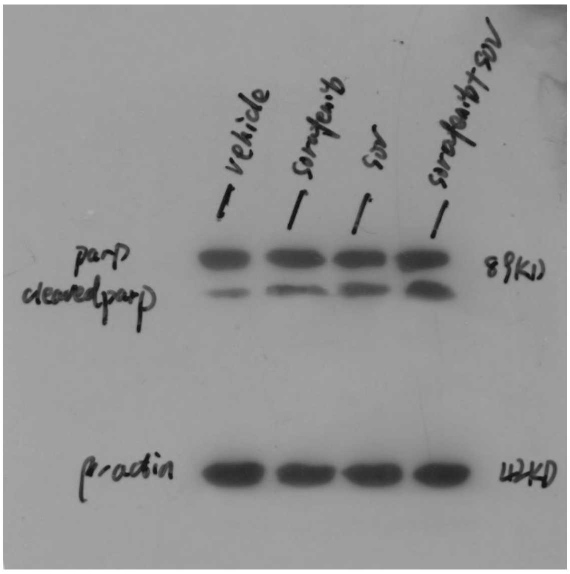
vehicle
sorafenib
SOV
sorafenib+SOV
parp
cleaved parp
89KD
β-actin
42KD
